# Inhibition of focal adhesion kinase 2 results in a macrophage polarization shift to M2 which attenuates local and systemic inflammation and reduces heterotopic ossification after polysystem extremity trauma

**DOI:** 10.3389/fimmu.2023.1280884

**Published:** 2023-12-05

**Authors:** Cassie J. Rowe, Uloma Nwaolu, Daniela Salinas, Jonathan Hong, Johanna Nunez, Jefferson L. Lansford, Conor F. McCarthy, Benjamin K. Potter, Benjamin H. Levi, Thomas A. Davis

**Affiliations:** ^1^ Cell Biology and Regenerative Medicine Program, Department of Surgery, Uniformed Services University, Bethesda, MD, United States; ^2^ Henry M. Jackson Foundation for the Advancement of Military Medicine, Inc., Bethesda, MD, United States; ^3^ Center for Organogenesis Research and Trauma, University of Texas Southwestern, Dallas, TX, United States

**Keywords:** heterotopic ossification, FAK2 inhibition, musculoskeletal trauma, tourniquet induced ischemia, amputation, inflammation, M1-M2 macrophage transition

## Abstract

**Introduction:**

Heterotopic ossification (HO) is a complex pathology often observed in combat injured casualties who have sustained severe, high energy polytraumatic extremity injuries. Once HO has developed, prophylactic therapies are limited outside of surgical excision. Tourniquet-induced ischemia injury (IR) exacerbates trauma-mediated musculoskeletal tissue injury, inflammation, osteogenic progenitor cell development and HO formation. Others have shown that focal adhesion kinase-2 (FAK2) plays a key role in regulating early inflammatory signaling events. Therefore, we hypothesized that targeting FAK2 prophylactically would mitigate extremity trauma induced IR inflammation and HO formation.

**Methods:**

We tested whether the continuous infusion of a FAK2 inhibitor (Defactinib, PF-573228; 6.94 µg/kg/min for 14 days) can mitigate ectopic bone formation (HO) using an established blast-related extremity injury model involving femoral fracture, quadriceps crush injury, three hours of tourniquet-induced limb ischemia, and hindlimb amputation through the fracture site. Tissue inflammation, infiltrating cells, osteogenic progenitor cell content were assessed at POD-7. Micro-computed tomography imaging was used to quantify mature HO at POD-56.

**Results:**

In comparison to vehicle control-treated rats, FAK2 administration resulted in no marked wound healing complications or weight loss. FAK2 treatment decreased HO by 43%. At POD-7, marked reductions in tissue proinflammatory gene expression and assayable osteogenic progenitor cells were measured, albeit no significant changes in expression patterns of angiogenic, chondrogenic and osteogenic genes. At the same timepoint, injured tissue from FAK-treated rats had fewer infiltrating cells. Additionally, gene expression analyses of tissue infiltrating cells resulted in a more measurable shift from an M1 inflammatory to an M2 anti-inflammatory macrophage phenotype in the FAK2 inhibitor-treated group.

**Discussion:**

Our findings suggest that FAK2 inhibition may be a novel strategy to dampen trauma-induced inflammation and attenuate HO in patients at high risk as a consequence of severe musculoskeletal polytrauma.

## Introduction

High energy blast-related trauma from improvised explosive devices is the leading cause of complicated multiple organ system injuries and death for servicemembers on the battlefield in recent military conflicts (1). Combat casualties with severe extremity wounds sustained in conflict (and once considered lethal) now survive due to advances in modern personal protective equipment technologies, far-forward positioning of critical care surgical teams, decreased medical evacuation times, and cutting-edge reconstructive surgery (2). While being fortunate to survive, these patients frequently experience complications in the convalescent period. A result of acute multi-mechanistic systemic responses to blast injury not commonly experienced in the civilian community, a delayed musculoskeletal pathologic condition that occurs with great frequency following wartime injuries is the formation of ectopic bone within soft-tissue, referred to as heterotopic ossification (HO) (3). Heterotopic ossification imposes morbidity resulting from impaired function and deceased use of affected limb secondary to joint contractures, tissue replacement and chronic pain (4).

Increasing evidence suggests hyperinflammation and immune dysregulation play a critical role in the onset and progression of the pathophysiology of complications induced by blast trauma (5). Nevertheless, the basis for this connection is not fully understood. The relationship between the immune system and trauma-induced HO formation is complex (6–11). However, an excessive proinflammatory response and hypoxia-mediated oxidative stress (iNOS, ROS) are considered to be the main mechanisms (10, 12, 13). Cytokine inflammatory pathways, mediated by activated monocytes-macrophages, are likely critical components of the microenvironmental cytokine milieu and play key roles in mediating HO formation following musculoskeletal trauma. Macrophage polarity plays a crucial role in the early innate immune response to trauma. During this process, accumulated macrophages transition from a polarized proinflammatory (M1) phenotype to an alternatively activated anti-inflammatory state (M2), a key *in vivo* cellular mechanism involved in mitigating inflammation, and promoting tissue repair and wound healing (14, 15)Pathogen-associated molecular patterns (PAMPs), damage-associated molecular patterns (DAMPs), and Th1 cell cytokines (IL-6, TNFα) activate resident macrophages to extend and augment inflammation, while Th2 cell cytokines (IL-4 and IL-13) polarize monocytes/macrophages to M2 macrophages releasing anti-inflammatory cytokines/chemokines (16, 17). M1/M2 polarization ratios in tissues have been suggested as potential prognostic factors to predict clinical outcome (18, 19).

The integrin-associated cytoplasmic protein tyrosine kinase, focal adhesion kinase-2 (FAK2), has been shown to be a key player of early signaling that orchestrates the initial development of focal adhesions. FAK2 has been shown to play important roles in the adherence of cells to the extracellular matrix (ECM) and cellular processes such cell adhesion, migration, proliferation, cytoskeletal organization, differentiation, and survival (20–23). FAK activity is regulated through extracellular integrin-ECM interactions and transmembrane receptors (G-protein-coupled, cytokine and growth factor receptors) at the plasma membrane), leading to downstream activation of phosphatidylinositol 3-kinase (PI3K) and AKT1, MAPK1/ERK2, MAPK3/ERK1 and MAP kinase signaling cascades (24). Together, these complex FAK-propagating signaling complexes are known to act as mechanosensors, responding to extracellular spatiotemporal environmental molecular and mechanical cues (25–28). Previously, focal adhesion kinase (FAK) and downstream YAP/TAZ have been identified as a link between mechanotransduction, hypoxic stress, inflammation, normal stem cell fate, and wound healing (29–32). A growing body of evidence suggest FAK overexpression is correlated with chronic inflammation which often leads to serious secondary complications and poor healing outcomes. The main immune cell implicated in in these FAK activation-mediated pathologies are macrophages. Chronic FAK-mediated proinflammatory molecule expression is promoted in endothelial cells by IL-1β and TNFα stimulation (29, 33, 34). Recent studies report that FAK signaling mediates LPS-induced inflammatory lung injury through increased transforming growth factor-β-activated kinase-1 (TAK1) and NFκB pathway activity (35) in macrophages. Moreover, FAK activation promotes lung fibrosis and myofibroblast formation following bleomycin lung injury (34). Others have reported that FAK signaling plays a critical role involved in the proliferation, migration, and proinflammatory activation of macrophages leading to chronic cardiac tissue inflammation in Chagas disease, diabetic cardiomyopathy, and other vascular diseases (36–38). Additionally, accumulating evidence demonstrates FAK signaling is overexpressed and activated in many tumor cells where it is critically involved with many diverse cell types (immune, stromal and tumor) and cellular/humoral processes (cytokines, chemokines, growth factors, ECM) within the tumor microenvironment that promote cell survival, proliferation, and metastasis resulting in poor prognosis (39, 40).

Increased FAK expression has been reported to increase angiogenesis and vascular permeability (41), two coupled processes essential for both normal and ectopic endochondral bone growth-osteogenesis (42–49). Intracellular FAK2 signaling regulates critical genes involved in the migration, proliferation, differentiation, survival, and osteogenesis of multipotent mesenchymal stromal cells (MSCs) and osteoblasts (23, 25, 46–48, 50, 51). Moreover, our initial studies have shown that FAK deletion in cells responsible for HO mitigates traumatic HO formation (52). Given that the resolution of inflammation is essential for proper wound healing along with bone and tissue regeneration, we hypothesized that the prophylactic inhibition of FAK2 with a pharmacologic inhibitor may attenuate inflammation in addition to ectopic bone formation in a rat poly-systemic trauma model (9, 10).

## Materials and methods

### Animals

Adult male (11-12-week old; 350 - 450 g) pathogen free Sprague Dawley rats (*Rattus norvegicus*) were obtained from Taconic Biosciences (Germantown, New York, USA). Animals were housed for a minimum of 7 days for acclimatization and quarantine purposes. All rats were pair-housed in individually ventilated cages and exposed to a 12-hour light/dark cycle, with free access to food (standard rodent chow) and water, under veterinary care and supervision. All experiments and animal care procedures for this research were approved by the Uniformed Services University Institutional Animal and Care and Use Committees (IACUC; Protocol # SUR-21-069). All activities were conducted in accordance with all applicable regulations, best practices pertaining to the use of animals in research, and the ARRIVE guidelines (53).

### Trauma-induced blast-related extremity injury and FAK2 inhibitor administration

**Figure 1:**
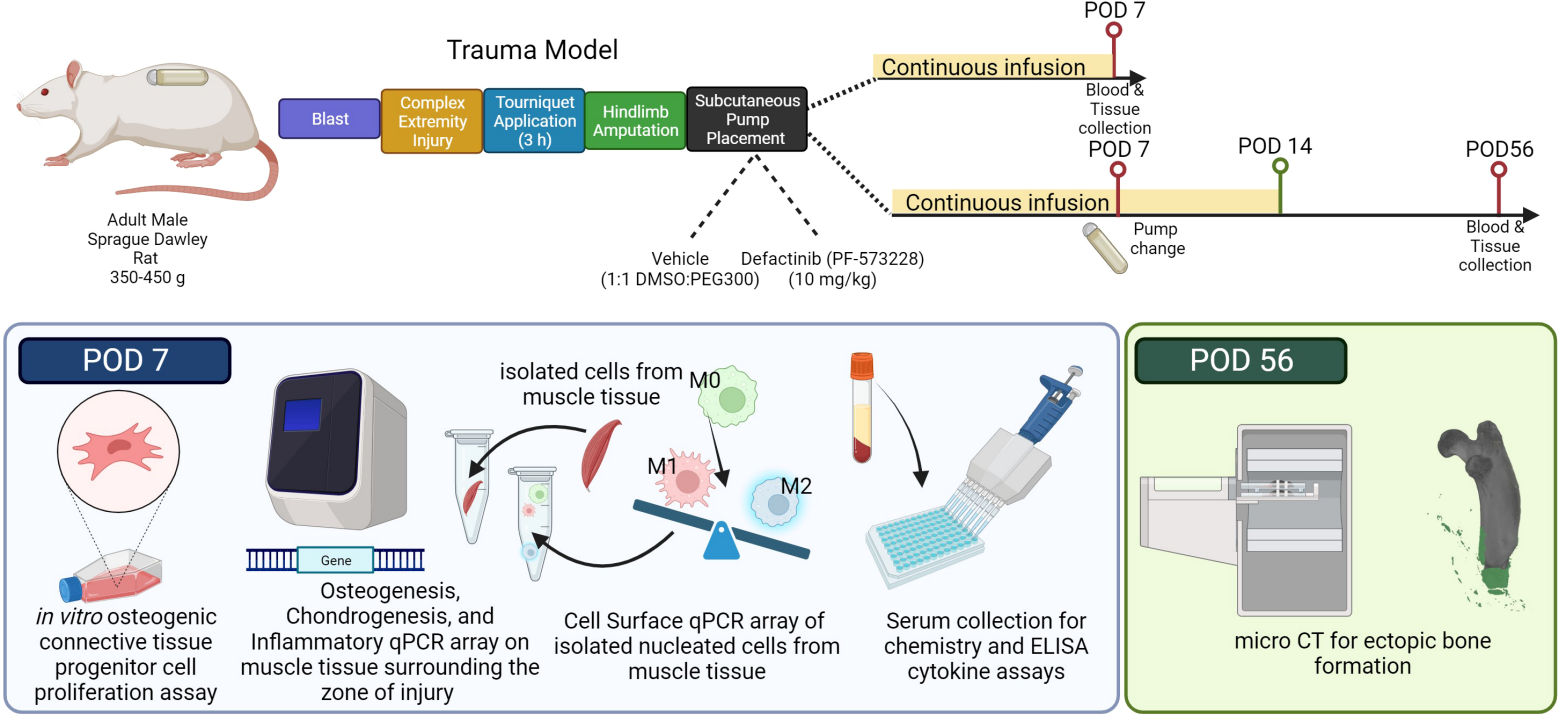
Illustration of the research study design and experimental approach. Adult male Sprague Dawley rats (350-450 g) underwent a sequential polysystem injury involving whole-body blast overpressure (120 kPa), a complex orthopaedic injury involving a mid-shaft femur fracture and medial quadriceps crush injury, followed by 180 min of tourniquet induced ischemia, and immediate hindlimb amputation. During the same anesthetic event, subcutaneous Alzet osmotic drug delivery pumps were implanted in the dorsum behind the scapulae containing either vehicle solution (1:1 DMSO to PEG300) or Defactinib (PF573228; mean pumping rate: 10 mg/kg/day; 6.94 µg/kg/min for 14 days). Cohorts of rats (n=5 per group for each timepoint) were euthanized on post-operative day (POD) 7 to assess inflammatory and early endochondral ossification signaling, or on POD-56 for morphometric analyses of ectopic bone formation. Schematic created with biorender.com web interface.

Rats (n=20) were subjected to the well-established blast-associated complex lower limb injury model, as previously described (**Figure 1**) (9, 54, 55). In brief, rats received sequentially a head-on whole-body blast overpressure exposure (120 kPa), a femoral fracture, soft tissue crush injury of the quadriceps, three hours of prolonged limb ischemia, and hindlimb amputation through the zone of injury (ZOI). A small incision and a subcutaneous pocket were formed, by blunt dissection, between the scapulae for implantation of an Alzet osmotic mini-pump (model 2ML1, DURECT Corporation, Cupertino, California, USA) for 7 days of continuous subcutaneous infusion of either vehicle control (n =10) or FAK2 inhibitor (Defactinib; PF-573228; 10 mg/kg/day (6.94 µg/kg/min; n =10)). The skin incision was closed with 5-0 nylon sutures. Pumps were surgically exchanged under isoflurane sedation at post-operative day (POD)-7 for a total of 14 continuous days of subcutaneous infusion. In some of these studies, a separate cohort of rats (n=6) were used to measure the concentration of PF-573228 in the serum using a liquid chromatography/tandem mass spectrometric (LC-MS/MS) system (Agilent 1200 series, Agilent Technologies, Inc., Santa Clara, California, USA). Prior to pump implantation, PF-573228 was dissolved in 50% dimethyl sulfoxide (DMSO) and 50% polyethylene glycol 300 (PEG 300) (56). Per manufacturer instructions, loaded pumps were placed in 0.9% NaCl at 37°C overnight to equilibrate before implantation. Animal health status was observed daily using IACUC approved pain score chart and body weights were recorded twice per week. Animals were euthanized on either POD-7 (n=10) or POD-56 (n=10) for follow-on morphometric or molecular analyses. Serum and muscle tissue obtained from age matched naive rats (n=5) served as the uninjured, healthy control.

### μCT imaging and quantification

Formalin fixed limbs collected at POD-56 were shipped to collaborators at the Center for Organogenesis Research and Trauma. Limbs were radiographically imaged using a MedisoUSA nanoScan PET/CT system (Arlington, Virginia, USA). The scanning parameters were max zoom with 720 helical projections with an x-ray power of 70 kV at 980 μA and an exposure time of 300 ms. Digitized images were reconstructed using the manufacturer’s program, Nucline nanoScan, with a resulting voxel size of 40 × 40 × 40 μm. Reconstructions of rat hindlimbs were created and quantified using Dragonfly ORS (Montreal, Quebec, Canada) software on representative means at 800 Hounsfield units. Semi-quantification analyses of ectopic bone formation were performed at the amputation injury site of the rat hindlimb by a blinded, skilled operator.

### Quantification of tissue infiltrating cells and osteogenic connective tissue progenitor cells

Following established protocols (45, 57, 58), skeletal muscle tissue located within the medial ZOI was aseptically collected and placed in sterile D-PBS with 100 U/mL penicillin (Invitrogen, Gaithersburg, MD). In brief, we removed fascia and fat from the tissue, minced it finely, and digested it in a solution of DMEM F-12 (Gibco, Billings, Montana, USA), Collagenase Types I and II, and Neutral Protease (Worthington, Lakewood, New Jersey, USA), for two hours. Cell suspensions were passed through a series of cell strainers (100, 70, 40 µm) to remove debris. Next, residual red blood cells were removed using ACK lysis buffer (Sigma-Aldrich, St. Louis, MO), washed with D-PBS, and centrifuged. Cell pellets were resuspended in osteogenic medium consisting of DMEM-F12, supplemented with 10% FBS, 100 nM of dexamethasone, 200 μM of ascorbic acid, 10 mM of glycerol 2-phosphate, and 100 U/mL penicillin (MilliporeSigma, Burlington, Massachusetts, USA). Cell yields and viability were determined using trypan blue dye exclusion. Cells (1-4 x 10^4^ per well; 6-well culture plate) were then plated in osteogenic medium for 10 days, with medium changes every 2 days. Culture wells were gently washed twice with D-PBS to remove non-adherent cells, air dried, fixed using 100% methanol and then stained with crystal violet. Entire plates were imaged using a fully-motorized Leica DMI6000B microscope (Leica, Wetzlar, Germany). Adjacent brightfield images from each well were stitched together using LAS X software (Leica, Wetzlar, Germany). Distinct CTP-Os (aggregates of > 50 cells) were manually counted as previously described (45, 59).

### Quantitative reverse transcription PCR gene analysis

Skeletal muscle (100 μg) and remaining infiltrating cells (1-4×10^6^) isolated within the ZOI surrounding the amputation site collected on POD-7 were flash frozen using liquid nitrogen, and stored at -80°C prior to RNA extraction. As previously optimized, a two-step RT-qPCR protocol was utilized to isolate RNA and transcribe cDNA (9, 55, 60) using the RNeasy Mini kit (Qiagen, Germantown, Maryland, USA) and the iScript Advanced cDNA synthesis kits (Bio-Rad, Hercules, California, USA). qPCR products were amplified using the SsoAdvanced ™ Universal SYBR Green Supermix, (Bio-Rad, Hercules, California, USA) according to the manufacturer’s instruction. The amplification was performed in 384-well plates using a QuantStudio real-time PCR system (QuantStudio 7 Pro, Applied Biosystems, Waltham, Massachusetts, USA). The data were acquired through ThermoFisher Connect software; the obtained mean Ct values were exported for statistical analysis. Muscle tissue collected from the medial quadriceps surrounding the ZOI was profiled for 120 gene targets consisting of chondrogenic, angiogenic, osteogenic, inflammatory, immune cell signaling, and early transcriptional activators for the assessment of early HO in injured muscle (**Supplementary Table 1**). Infiltrating cells harvested from muscle tissue were profiled for a panel of 40 gene for cell surface markers expressed on immune cells (**Supplementary Table 2**). Each gene array contained optimal housekeeping genes and assay controls (Bio-Rad, Hercules, California, USA).

### Serum IL-6 and IL-13 measurements

The systemic concentration of Th1 interleukin-6 (IL-6), and Th2 interleukin-13 (IL-13) in serum specimens collected from POD-7 animals were analyzed using commercial protein arrays (Meso Scale Diagnostics, Rockville, Maryland, USA) according to the manufacturer’s instructions. Data acquisition was performed using a Meso Sector S600 (Meso Scale Diagnostics, Rockville, Maryland, USA) and quantitative results were generated using Methodical Mind software (version MMPR 1.0.27; Meso Scale Diagnostics, Rockville, Maryland, USA).

### Data analysis and statistics

All data was curated using GraphPad Prism (version 9.5.1, San Diego, California, USA). Data analysis was performed using either SPSS (version 28.0.1.0 (142); SPSS Inc., Chicago, Illinois, USA) or Graph Pad Prism. Outliers, as necessary, were removed using the Rout method (Q=1%). Data are presented as mean ± SEM. Statistically significant comparisons were denoted as follows: * for p<0.05, ** for p<0.01, *** for p<0.001, or **** for p<0.0001, with brackets identifying the source of significance.

#### Micro-CT

A two-tailed unpaired t-test with a 95% confidence level was conducted using GraphPad Prism to compute the differences in total ectopic bone volume at the ZOI of the injured limb between the vehicle and PF-573228-treated animals.

#### Connective tissue progenitor assay

Each assessment, including the number of isolated cells, frequency of CTP-Os, and number of CTP-Os per gram of muscle, was evaluated with GraphPad Prism using a two-tailed unpaired t-test with a 95% confidence level. The t-test was conducted to determine statistical differences between cells or colonies derived from muscle tissue surrounding the ZOI in either vehicle or PF-573228-treated animals. Treatment effect size was calculated using Glass’s delta (GΔ) formula when variances between groups were significantly different (61–63). Ranking of effect size: small effect = 0.2 medium effect = 0.5, large effect > 0.8. A GΔ lower cutoff threshold of 0.75 was chosen to indicate significant effect size.

#### Gene expression

Relative expression (2^-ΔCt^) was calculated for naïve and injured samples using the optimal normalization strategy (55). A cycle threshold (Ct) level tagged as “undetermined” by the Cloud Connect software (Thermo Fisher Scientific) for any particular gene was considered not expressed. However, to explore changes in genes not expressed in naïve conditions but expressed following injury or vice versa, a Ct value of 40 was imputed for calculation purposes (64). All statistical analyses were conducted on ΔCt values. To assess for significant differences in relative gene expression between muscle tissue samples obtained from naïve or injured (vehicle- and PF-573228-treated) animals, we compared the ΔCt values using a one-way ANOVA on SPSS software (version 28.0.1.0 (142); SPSS Inc., Chicago, IL, USA), with α=0.05. A Tukey-Kramer *post-hoc* analysis test was utilized to determine the source of the significance.

#### Serum cytokine and clinical chemistry measurements

A one-way ANOVA with Welch statistic, on GraphPad Prism, was used to assess significant differences between serum levels of individual analytes collected from naïve controls or injured animals on post-operative day 7. Dunnett’s T3 multiple comparisons test was used to determine the source of significance with α=0.05.

## Results

### FAK2 inhibitor attenuates ectopic bone formation

**Figure 2:**
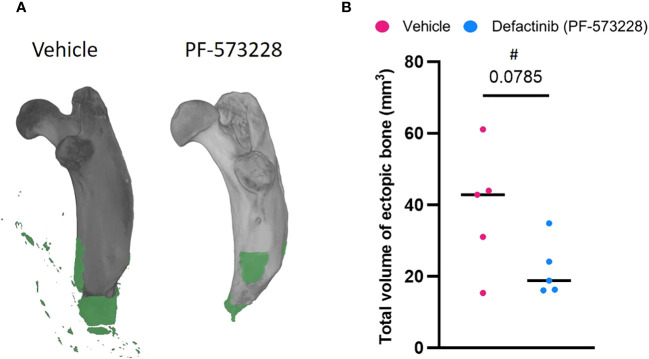
FAK2 inhibition mitigates trauma-induced heterotopic ossification (HO) formation. Micro-computed tomography scans were performed on vehicle- and PF573228-treated rats following sequential polysystem injury at week 8. **(A)** Total new bone was determined using Dragonfly software to identify the difference between new bone (green) and naïve bone (grey). **(B)** Quantitative measure of ectopic bone volume. Unpaired t-test analyses revealed a trend (p=0.0785) towards a reduced formation of ectopic bone at the amputation site in the PF573228-treated injured rats (n=5; 10 mg/kg/day; 6.94 µg/kg/min for 14 days) compared to the vehicle-treated injured rats (n=5). Data presented as mean values ± SEM. ^#^ indicates a large effect size (GΔ = 1.05).

A continuous subcutaneous infusion of the FAK2 inhibitor Defactinib (PF-573228) at 10 mg/kg/day (6.94 µg/kg/min) resulted in a marked reduction (43%) in the amount of new bone formation at 8-weeks post trauma (**Figure 2**) when compared to the vehicle control group (22.03 ± 16.95 mm^3^ *vs* 38.88 ± 7.85 mm^3^ ectopic bone; p=0.0785). The independent samples t-test analysis revealed no significant difference in HO volume between vehicle-treated (M = 38.88, SD = 16.95, CI = 29.13-48.63) and FAK2-treated (M = 22.03, SD = 7.85, 95% CI 0.9-43.08)), t = 2.017, p = 0.0785. The effect size, as measured by GΔ = 1.05, indicating a large effect.

Reconstructions from µCT analysis showed a clear development of new bone tissue proximal to the amputation site and within the surrounding soft-tissue. Histological evaluation confirmed the findings from the radiographic analysis (data not shown). LC-MS/MS determinations measurements showed a continuous intravenous dose of PF-573228 at 10 mg/kg/day (6.94 µg/kg/min) resulted in a serum concentration of 0.25 ± 0.17 ug/mL on day-3 (n=3). To investigate the effectiveness of short-term FAK2 inhibitor treatment without major adverse consequences, we monitored health status and body weight during the first 4 weeks of treatment. Compared to the vehicle-treated group, the PF-573228-treated group had slightly decreased weight gain, but there were no apparent differences in pain scores. Similar serum clinical chemistry measurements were detected between the two groups (**Supplementary Figure 1**) on POD-7. However, a significantly marked decrease of AST (p=0.028) was detected in the PF-573228-treated group compared to the vehicle. Compared to historical findings using this trauma model, no evidence of surgical site infections and/or adverse gross wound healing complications-wound breakdown resulting in delayed wound healing were noted in either group.

### FAK2 inhibition suppresses trauma-mediated inflammatory, chondro-angio-osteogenic signaling that promotes osteogenic progenitor cells growth and HO formation in traumatized muscle

**Figure 3:**
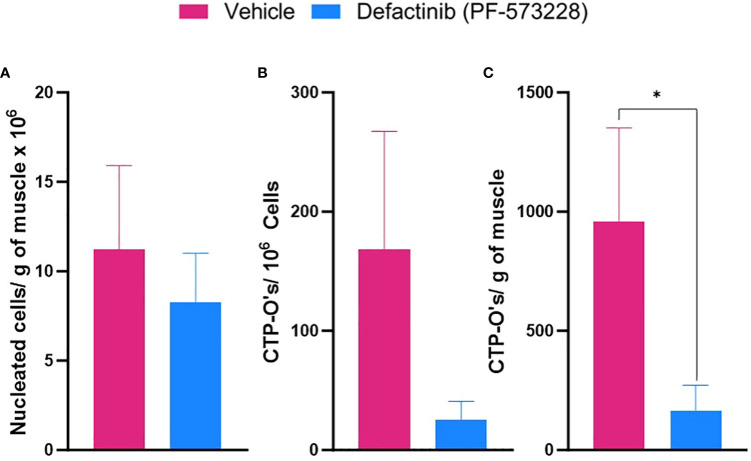
FAK2 inhibition attenuates injured muscle tissue cellular infiltration and osteogenic connective tissue progenitor colony-forming cell activity (CTP-O). Following blast-related polytraumatic extremity injury and 6 days of vehicle or FAK2 inhibitor treatment (PF573228, 10 mg/kg/day, 6.94 µg/kg/min), muscle tissue surrounding the amputation site was harvested on POD-7. Viable nucleated cells were isolated using collagenase digestion, counted and plated in osteogenic culture media. **(A)** Number of nucleated cells isolated per gram of muscle. **(B)** Number of assayable CTP-Os per 1 x 10^6^ plated cells. **(C)** Quantification of number of CTP-Os per gram of tissue by crystal violet staining after 10 days of *in vitro* culture in osteogenic media. Visible macroscopic colonies containing > 50 cells were enumerated. Data represents mean values ± SEM. * indicates p <0.05.

Inflammation is a major driver of heterotopic endochondral ossification process following tissue trauma. We therefore hypothesized, FAK2 inhibition may be a useful therapeutic to dampen the local early immune response induced by acute musculoskeletal trauma. To this end we investigated, at the peak of the tissue inflammatory response (POD-7), the level of inflammatory cell infiltration, presence of assayable connective tissue osteogenic progenitor cells (CTP-Os), and expression of levels of inflammatory, chondrogenic, angiogenic and osteogenic genes within muscle tissue obtained from the site of injury. The frequency and total number of isolated nucleated cells per gram of injured muscle tissue (**Figures 3A, B**) in the PF-573228-treated group (8.27×10^6^ ± 2.76×10^6^, n=5) was modestly reduced (p = 0.614) in comparison to the vehicle control group (11.21×10^6^ ± 4.7×10^6^; n =4). FAK2 inhibition treatment reduced the number assayable CTP-O colony forming progenitor cells (**Figure 3C**) by 86% when compared to vehicle control treatment The independent samples t-test analysis revealed a significant difference in number of tissue resident CTP-Os per gram of tissue between vehicle-treated (M = 1194, SD = 985, 95% CI = 636-4867) and FAK2-treated (M = 166, SD = 238, 95% CI = 24-850)), t = 2.290, p = .05). The effect size GΔ = 1.04, indicating a large effect.

**Figure 4:**
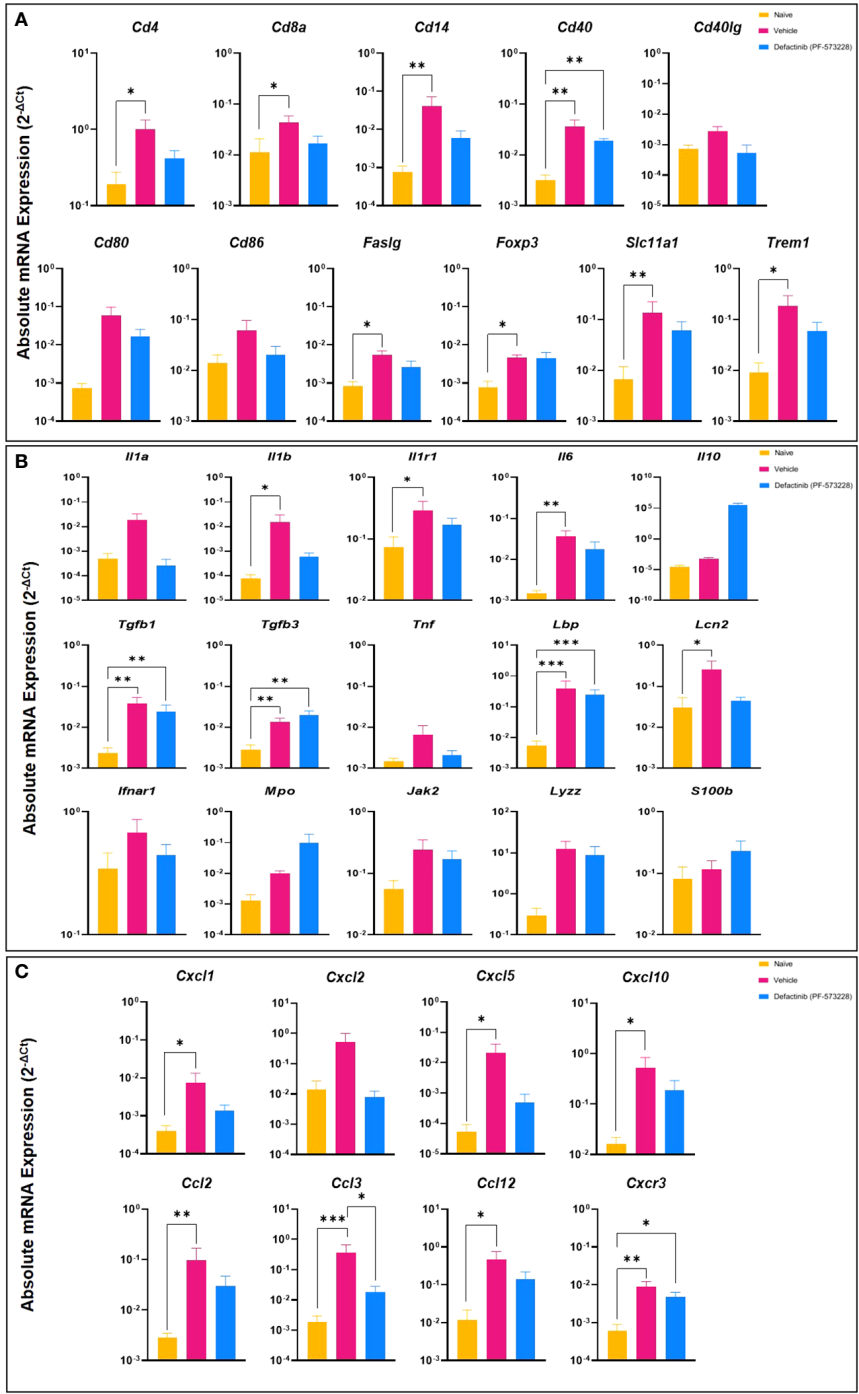
Levels of inflammatory mediators in muscle tissue collected from the amputation site at POD-7 from rats treated with FAK2 inhibitor (PF573228;10 mg/kg/day; 10 mg/kg/day, 6.94 µg/kg/min)), vehicle control, or healthy muscle in age-matched controls. Panel **(A)** cell surface markers specific to immune cells; Panel **(B)** inflammatory signaling molecules and cytokines; Panel **(C)** chemokines. Relative expression (2^-ΔCt^) was calculated using an optimal normalization strategy. One-way ANOVAs for each gene were conducted on ΔCt values to determine treatment effects. Tukey-Kramer *post-hoc* analyses were utilized to determine the source of the significance. Data represents mean relative expression values ± SEM. * indicates p < 0.05, **indicates p < 0.01 and *** indicates p < 0.001.

To further characterize the impact of FAK2 inhibition in our HO trauma model, we assessed transcript expression level of genes that regulate the early development of endochondral bone formation. As shown in **Figures 4A-C**, blast/extremity injury resulted in significant increases in expression levels of cell surface markers specific for immune cells (*Cd4*, *Cd14*, *Faslg*, *Foxp3*, *Slc11a1*, and *Trem1*), inflammatory signaling molecules and cytokines (*Il1b*, *Il1r1*, *Il6*, and *Lcn2*), and several chemokines (*Cxcl5*, *Cxcl10*, *Cxcl2*, and *Ccl12*). This significant reduction in inflammatory transcripts following PF573228 treatment corresponds with the measured reduction in the number of muscle infiltrating cells isolated at POD-7. Additionally, we found that FAK2 inhibition had a modest suppressive effect on a number of osteogenic genes (*Atf3*, *Jun*, *Socs3*, and *Traf6*) while upregulating the expression of a few chondrogenic genes including *Acan*, *Comp*, and *Sox9* (**Supplementary Figures 2A, B**). Overall, short-term FAK2 inhibition had no impact on the expression of a number of angiogenic, toll-like receptors, adhesion/matrix proteins, or apoptotic-related gene transcripts (**Supplementary Figures 2C-H**).

### Increased M2 macrophage tissue infiltration is associated with attenuated inflammation and HO formation

**Figure 5:**
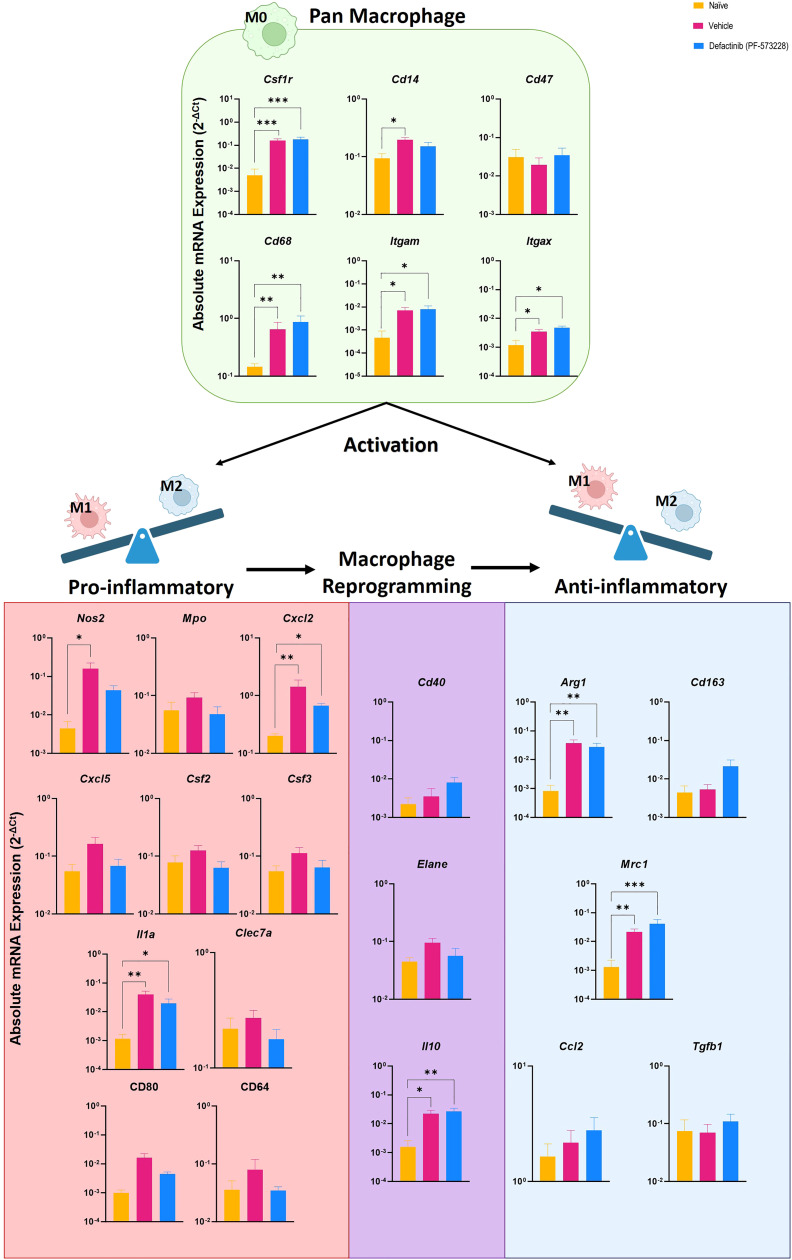
Level of pan-macrophage, classic proinflammatory M1 and alternative activated anti-inflammatory M2 gene markers expressed in cellular infiltrates collected from injured muscle tissue at POD-7 from rats treated with the FAK2 inhibitor (PF-573228,10 mg/kg/day, 6.94 µg/kg/min), vehicle control and in healthy muscle from age-matched controls. Relative expression (2^-ΔCt^) was calculated using an optimal normalization strategy. One-way ANOVAs for each gene were conducted on ΔCt values to determine treatment effects. Tukey-Kramer *post-hoc* analyses were utilized to determine the source of the significance. Data represents mean relative expression values ± SEM. * indicates p < 0.05, **indicates p < 0.01 ands *** indicates p < 0.001.

**Figure 6:**
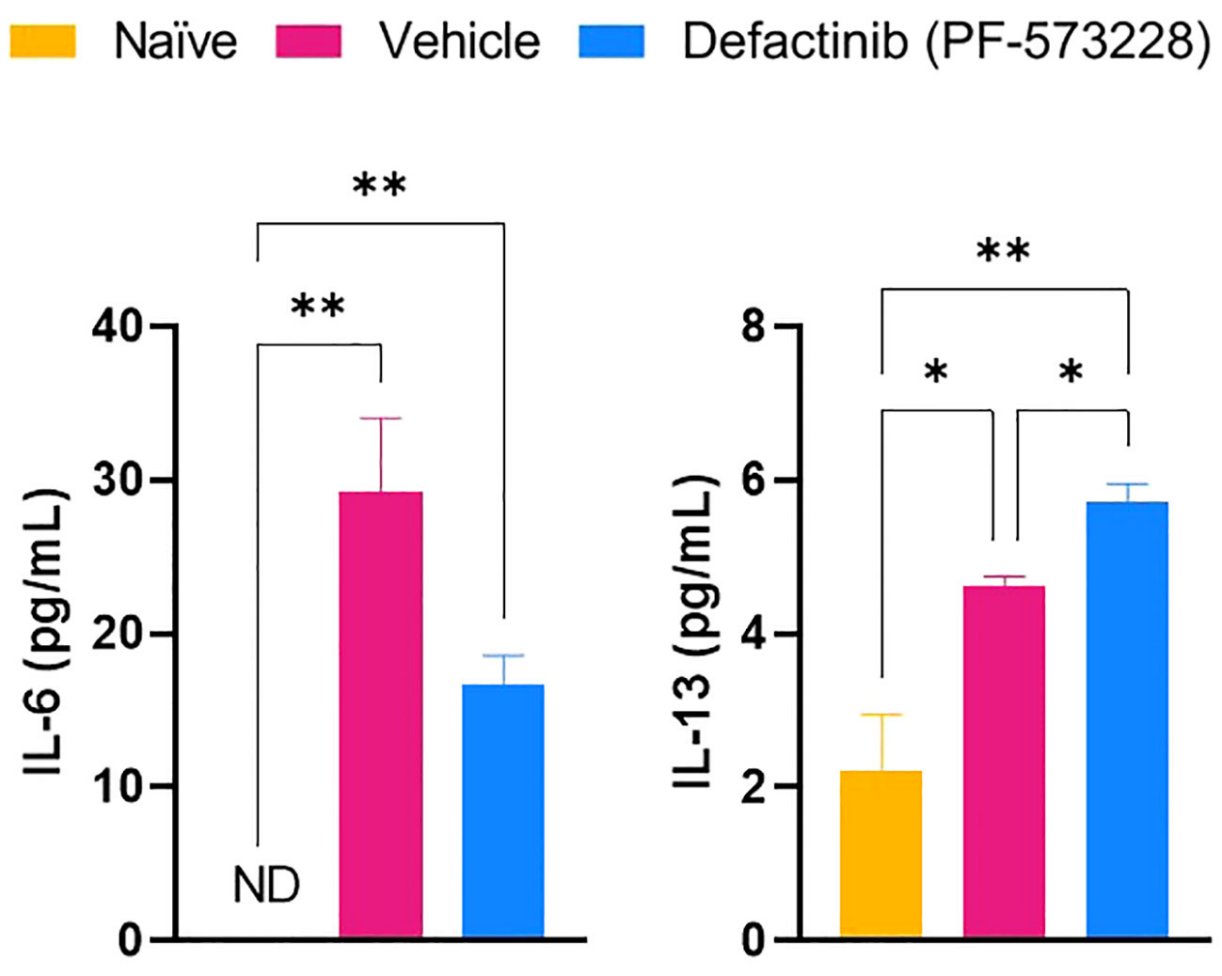
The effect of FAK2 inhibitor (PF-573228; 10 mg/kg/day, 6.94 µg/kg/min) on systemic proinflammatory IL-6 and anti-inflammatory IL-13 secretion levels at POD-7. Results from non-injured naïve rats, vehicle control-treated rats, and FAK2 inhibitor-treated rats are expressed as mean value ± SEM, n = 25. * indicates p<0.05 and ** indicates p<0.01.

To test whether FAK2 signaling also regulates macrophage transition *in vivo*, we explored the association of M1 and M2 macrophage cell composition by transcriptomic markers in cellular infiltrates collected from injured-regenerative muscle tissue from vehicle control-treated rats (**Figure 5**). Infiltrating cells collected from FAK2 inhibitor and vehicle-treated rats on POD-7 had very similar expression levels of pan-macrophage markers (*Csf1r, Cd1, Cd47, CD68, Itgam, Itgax*). Cells from vehicle control-treated rats had relatively higher expression levels of proinflammatory M1 molecular signature markers (*Nos2, Mpo, Cxcl2, Cxcl5, Csf2, Csf3, Il1a, Clec7a, Cd80, Cd64*) of traditional-classic activated macrophage phenotype. However, muscle infiltrating cells obtained from FAK2 inhibitor-treated rats showed relatively similar or higher expression levels of anti-inflammatory M2 markers (*Arg1*, *CD163*, *Mrc1*, *Ccl2*, *Tgfb1*) consistent with alternatively activated macrophage functional phenotype. The gene expression levels of three activation markers (*Cd40, Elane, Il10*) in both cell populations was similar and have been depicted as transitional repolarization M1-M2 signatures. Taken together, these results based on mRNA expression analysis, indicate that infiltrating cells differentiate and polarize more towards an M2 phenotype, with decreased M1 and increased M2 macrophage phenotype cytokine expression, in response to FAK2 inhibition. Notably, serum IL-6 and IL-13 levels (**Figure 6**), which are involved with M1 programming (IL-6) and M2 polarization (IL-13), were decreased and increased, respectively, in the FAK2 inhibitor treatment group when compared to levels in the vehicle control group.

## Discussion

Combat-related wounds are normally associated with a persistent local and systemic inflammatory response that often result in delayed wound closure and healing (65, 66). Previous research has indicated that prolonged, elevated inflammation at the site of musculoskeletal trauma is one of the key contributors to impaired wound healing and development of heterotopic ossification (HO) (7, 8, 44, 45, 57, 59, 67–70). As an important regulator of cytokine and integrin-mediated signaling, FAK2 is involved in various cellular processes including cell adhesion, motility, proliferation, osteogenesis and bone homeostasis, through its kinase activity and scaffolding functions under steady and pathologic states (22, 23, 71–74). Tourniquet-induced ischemia-related injury (IR) exacerbates trauma-mediated musculoskeletal tissue injury, inflammation, osteogenic progenitor cell development and HO formation. Others have shown that FAK2 plays a key role in regulating early inflammatory signaling events. Therefore, we hypothesized that targeting FAK2 prophylactically may mitigate extremity trauma induced inflammation and HO formation using a validated extremity trauma model.

In this study, we found that early administration of PF-573228, an ATP analog that acts to inhibit FAK kinase activity, following polysystem extremity trauma involving blast blunted inflammation, osteogenic gene signaling, and the recruitment and growth of osteogenic progenitor cells at the site of injury resulting in reduced HO formation. We also provide gene signature evidence that macrophages with the M1 phenotype accumulate in the in the injured skeletal muscle. *In vivo* FAK2 signaling inhibition, promotes the differentiation and polarization switch of infiltrating macrophages into a M2 phenotype program, possibly contributing to earlier inflammation resolution, tissue repair, and wound healing (75). These results emphasize the interaction FAK2 signaling plays in regulating distinct cellular and molecular mechanisms post-traumatic injury. Therefore, inhibiting FAK2 signaling *in vivo* systemically or with topical application of pharmacological inhibitors might offer a new target attractive strategy for limiting inflammation and inhibiting HO.

Earlier studies demonstrate FAK adhesion-dependent signaling is critical for new bone formation, as it promotes osteoblast progenitor proliferation, differentiation, and mechanotransduction through enhancement of Akt, mTOR and WNT signaling (22, 23, 74, 76, 77). MSCs engineered to over-express FAK exhibit a marked increase in their osteogenic potential *in vitro*. FAK promotes the expression of osteoblast phenotypic markers as well as *in vitro* osteogenesis in human mesenchymal stromal cells (hMSCs). Interestingly, Kim et al. (73) showed in transgenic FAK−/− mice, osteogenic progenitors were able to migrate to the site of skeletal trauma, however a significant delay in osteoblast differentiation and matrix formation was measured. Further, Qi et al. (23) report that FAK-deficient osteoprogenitor cells have lessened proliferation and significantly reduced mammalian/mechanistic target of rapamycin complex 1 (mTORC1) signaling. Moreover, we previously demonstrated the robust efficacy of rapamycin in inhibiting blast trauma-induced HO (45). In this study, we show that inhibition of FAK2 during skeletal muscle regeneration results in reduced inflammation (IL-6), inflammatory cell accumulation, and expression of *Runx2* and *Osterix* which are considered master transcription factors in regulating osteogenic differentiation of MSCs and regulation of bone matrix genes (72, 78). Mitogen-activated protein kinase (MAPK) and/or extracellular signal-regulated kinase (ERK) pathway activation of Runx2 triggers a cascade of the downstream osteoblast-related genes to promote the differentiation of osteoblasts for bone matrix synthesis and mineralization (79). Taken together, our findings are consistent with studies that report the loss or inhibition of FAK2 impairs osteoprogenitor cell differentiation (46, 73). More specifically, revealing that attenuated inflammation mediated FAK2 signaling may suppress the activation, proliferation, and/or *in vivo* migration of endogenous MSCs and osteogenic progenitor cells resulting in reduced ectopic bone formation following trauma. With regard to the muscle gene expression, blast/extremity injury resulted in significant increases in expression levels of cell surface markers specific for immune cells and inflammatory signaling molecules (interleukins, chemokines) as compared to naive muscle. In contrast, in the FAK2-treated muscle, there was a modest suppressive effect (reduction) in transcript levels of the same markers; even though these levels were elevated above naive levels these elevations were non- significant. The loss of significant elevations in gene expression of inflammatory mediators and cell surface markers is an important finding.

Macrophages play key roles in the inflammation, proliferation, and remodeling phases of wound healing. Throughout the wound healing process, converging inflammatory stimuli in the cellular microenvironment have been shown to facilitate the transition of local macrophages from a pro-inflammatory (M1-like phenotype) to anti-inflammatory (M2-like phenotype) state (14, 15). Robust inflammatory cell infiltration, macrophage activation as well as inflammatory and osteogenic cytokine/chemokine release contribute the microenvironmental niche that contributes to HO formation (80). Macrophages present in virtually all tissues are functionally regulated by their surrounding micro-environment stimuli (81).

Recently, evidence has indicated that the proinflammatory (M1) macrophage contributes to the hypoxia microenvironment and an excessive inflammatory, angio-chondro-osteogenic cytokine milieu which drives the differentiation of multipotent progenitors (MPPs) found in injured skeletal muscle into bone (80, 82). On POD-7, we detected elevated levels of pro-inflammatory cytokines along with cellular dysregulation consistent with increased reactive oxygen species (ROS) levels. Our findings demonstrate the proinflammatory M1 macrophage is the prominent inflammatory cell in traumatized muscle at the point when early angio-chondro-osteogenic signaling and progenitor cell activity is being established. In comparison, FAK2 inhibitor treatment (PF-573228) resulted in reduced cellular infiltration. Comparative gene expression studies and osteogenic progenitor cell assays of collected infiltrating cell populations, revealed infiltrating cells collected after FAK2 inhibition expressed less proinflammatory and angio-chondro-osteogenic genes with lesser prevalence of osteogenic colony-cells, respectively.

The signaling events underlying the crosstalk between M1-M2 macrophage phenotypic states, other immune cells, and MPPs within the osteogenic niche in the absence of FAK2 inhibition or before and after systemic macrophage depletion are unclear. M2 macrophages express IL-10, arginase I, and chemokines (TGFβ, IL-13), which play crucial roles in the resolution of inflammation, wound healing, and tissue remodeling (14, 15). M2, but not M1, macrophages have been reported to have anti-calcifying activity in vascular smooth muscle cell (VSMC) calcification (83),whereas others have reported in models of healing and sepsis the importance of wound macrophage transition from the predominantly pro-inflammatory phenotype present early post-injury to the anti-inflammatory phenotype to accelerate wound healing (84). On the other hand, Abshire et al. (85) report that loss of FAK expression results in reduced polarization, chemotaxis to CSF-1 (G-CSF) and recruitment to sites of inflammation (85). Likewise, Owen and colleagues (86) show bone marrow-derived macrophages from FAK−/− mice exhibit reduced chemotaxis toward CSF-1. A wide array of cell types produce CSF-1, a cytokine/growth factor, which possesses both autocrine and paracrine roles. It promotes the migration, proliferation, functionality, and survival of macrophages, while fostering M2 macrophage polarization (87). Our results bring new indications for the role of macrophages and their activation status in the initiation and development of HO. Future studies evaluating impact of FAK2 silence using knockout rats in this model may provide additional important information and strengthen our hypotheses.

Previous studies assessing wound healing and various pathologies demonstrated the mechanisms regulating macrophage polarization are diverse and complex. The mechanisms also involve the concurrent regulation of various cytokines, chemokines, and signaling pathways, to include PI3K/Akt, TLR4/NF-κB, mTOR, JAK/STAT, TGF-β/Smads, JNK/c-My, PPARγ and others (88) Zhang et al. (89) demonstrated that the promotion of macrophage polarization from M1 to M2 is facilitated by MCP-induced protein 1 through the suppression of the JNK/c-Myc signaling pathway. While our studies suggest a possible involvement of FAK2 in macrophage polarization regulation. Nonetheless, the function of FAK2 expression in regulating critical transcription pathways that control macrophage function and activity, has not yet been established in our wound healing model of acute musculoskeletal trauma. In summary, our transcriptomic studies have highlighted the necessity to further characterize the full spectrum of phenotypic changes of recruited-resident macrophage populations as these activated macrophages most likely fall into various states of polarization between M1 and M2.

This study has a number of limitations. First, PF-573228 inhibits FAK activity as well as the activity of other related kinases. Second, PYK2, a close paralogue to FAK has been shown to functionally compensate for loss of FAK (90, 91), albeit 50- to 250-fold less selective for FAK than PYKA (92). In oncological and chronic disease applications dual FAK/PYKA inhibition has been shown to be more efficacious (93). Hence, future therapeutic studies targeting both kinases are warranted. Third, while many of our results reached statistical significance, due to the increased biological variability encountered in a complex injury model, variance in results was often high, affecting the ability to detect statistical significance. Larger sample sizes for future studies should therefore be considered. Alternatively, we extended the HO analysis to calculation of mean effect size which was large, albeit the power of the experiment and sample size (number of animals per group) using a two-sample unpaired t-test was not able to detect this effect. The 95% Confidence Intervals support our conclusions that the mean sizes are large. While the measured differences between treatment groups may not be statistically significant (strong enough to reject the Null hypothesis), we suggest the strength of these results are highly clinically relevant for clinical patient care applications. Fourth, further work is required to characterize the impact of FAK2 inhibition in promoting the M1-M2 transition and identifying the biological variation of endogenous and tissue infiltrating cells. A comprehensive assessment using immunohistochemistry, single-cell RNA sequencing and multiparameter flow cytometry may provide additional critical insight. This approach could reveal the presence and activation status of regulatory/reparative cell subsets, detection of rare cell types in complex tissues, and highlight opportunities for further treatment refinement. Fifth, we did not investigate potential sex differences in this study; all the animals utilized were males. Notably, acute inflammatory responses differ between male and female rats, as well as in humans. Prior reports have shown that males are more susceptible to traumatic injury (94, 95). However, the utilization of only male rats in this study is justified, considering that fewer than 3% of severely combat-injured service members in recent conflicts were female (96). Last but not least, it is critical to transition and expand this knowledge to large animal model investigations.

Prolonged inflammation is a known critical driver of trauma-induced ectopic bone formation. In this study, we show continuous administration of the FAK2 inhibitor PF-573228 for 14-days post injury dampened tissue inflammation, suppressed osteogenic signaling and development, and promoted M1-M2 macrophage polarization thought to relieve inflammation and promote accelerated tissue repair resulting in reduced progenitor cell activity and subsequent HO formation.

## Data availability statement

The datasets used and/or analyzed during the current study are available from the corresponding author on reasonable request.

## Ethics statement

The animal study was approved by Uniformed Services University Institutional Animal and Care and Use Committees (IACUC; Protocol # SUR-21-069). The study was conducted in accordance with the local legislation and institutional requirements.

## Author contributions

CR: Data curation, Formal analysis, Investigation, Methodology, Project administration, Validation, Writing – review & editing, Visualization. UN: Data curation, Investigation, Methodology, Writing – review & editing. DS: Writing – review & editing, Data curation, Investigation, Methodology. JH: Investigation, Writing – review & editing, Methodology. JN: Investigation, Writing – review & editing. JL: Investigation, Writing – review & editing. CM: Investigation, Writing – review & editing. BP: Writing – review & editing, Conceptualization, Funding acquisition. BL: Investigation, Methodology, Writing – review & editing, Conceptualization, Funding acquisition. TD: Investigation, Methodology, Writing – review & editing, Conceptualization, Data curation, Formal analysis, Funding acquisition, Project administration, Supervision, Validation, Writing – original draft.

## Supplementary material

Supplementary Figure 1 Treatment with PF-573228 results in no change in serum chemistry analytes at POD-7. Quantification of rat serum chemistry of naïve, or blast-extremity injury animals treated with vehicle solution or continuous infusion of PF573228 FAK inhibitor for 7 days. Statistical significance was calculated using a one-way ANOVA with Turkey *post-hoc* was used to test for statistical significance. Data are represented as mean values ± SEM. * indicates p < 0.05, ** indicates p < 0.01, *** indicates p < 0.001, **** indicates p < 0.0001).

Supplementary Figure 2 Relative expression levels of early signaling genes involved in the development of heterotopic ossification including **(A)** osteogenesis **(B)** chondrogenesis **(C)** angiogenesis **(D)** adhesion and integrins **(E)** matrix metalloproteinases and tissue inhibitors of metalloproteinases **(F)** toll-like receptors **(G)** transcriptional activators and cytoplasmic protein kinases and **(H)** apoptosis. For this experiment, muscle tissue was collected from the amputation site at POD-7 from rats treated with FAK2 inhibitor (PF-573228;10 mg/kg/day; 10 mg/kg/day, 6.94 µg/kg/min)), vehicle controls, or in healthy muscle from age-matched controls. Relative expression (2^-ΔCt^) was calculated using an optimal normalization strategy. One-way ANOVAs for each gene were conducted on ΔCt values to determine treatment effects. Tukey-Kramer *post-hoc* analyses were utilized to determine the source of the significance. Data represents mean relative expression values ± SEM. * indicates p < 0.05, **indicates p < 0.01, *** indicates p < 0.001 and **** indicates p < 0.0001.
