# Supplementary figures and images for: Inhibition of focal adhesion kinase 2 results in a macrophage polarization shift to M2 which attenuates local and systemic inflammation and reduces heterotopic ossification after polysystem extremity trauma
Source: Front Immunol. 2023 Dec 5;14:1280884. doi: 10.3389/fimmu.2023.1280884 (PMC10728492; doi:10.3389/fimmu.2023.1280884)

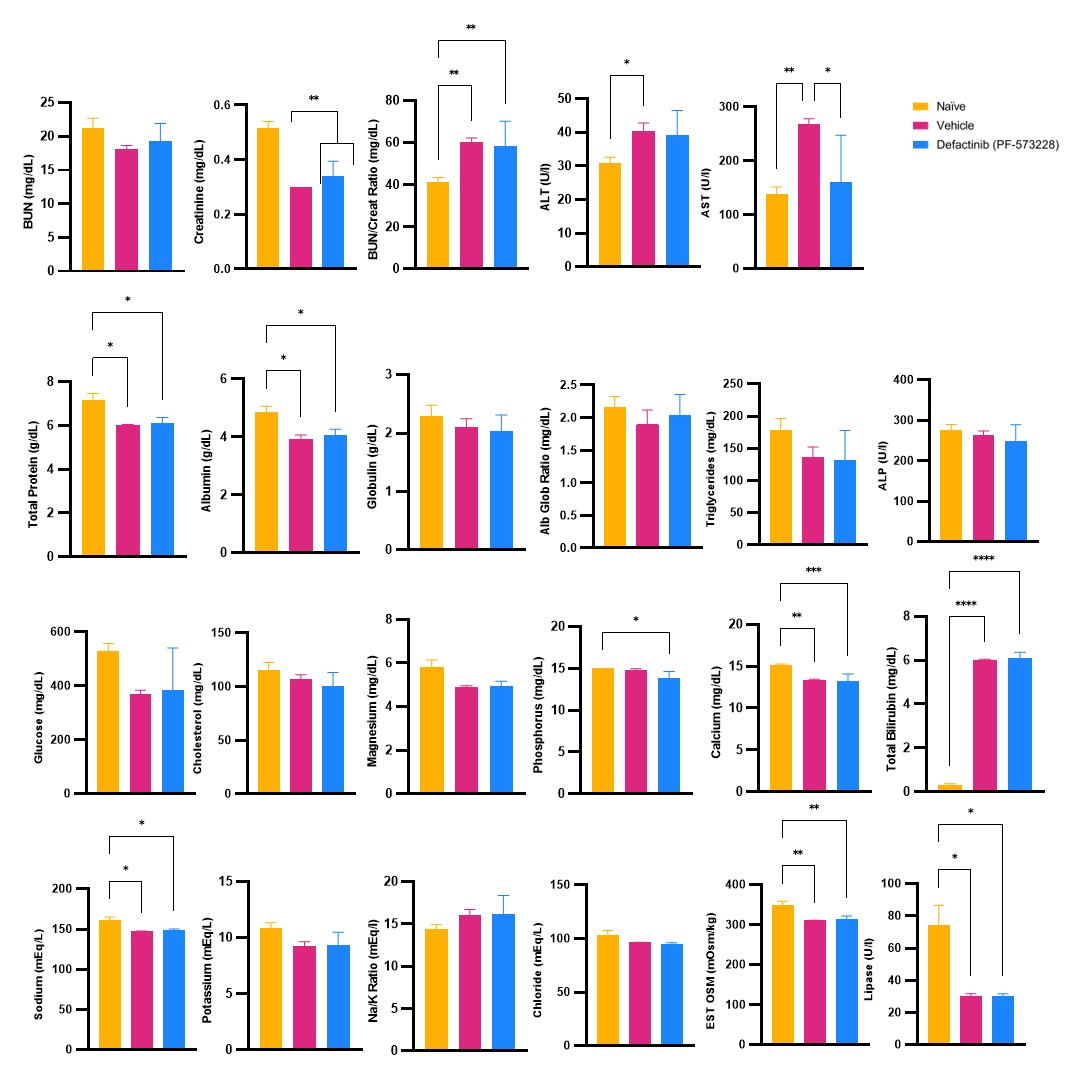

Supplement: Supplementary Figure 1 — Treatment with PF-573228 results in no change in serum chemistry analytes at POD-7. Quantification of rat serum chemistry of naïve, or blast-extremity injury animals treated with vehicle solution or continuous infusion of PF573228 FAK inhibitor for 7 days. Statistical significance was calculated using a one-way ANOVA with Turkey post-hoc was used to test for statistical significance. Data are represented as mean values ± SEM. * indicates p < 0.05, ** indicates p < 0.01, *** indicates p < 0.001, **** indicates p < 0.0001). [file Image_1.jpeg]
