# Supplementary material for: Inhibition of focal adhesion kinase 2 results in a macrophage polarization shift to M2 which attenuates local and systemic inflammation and reduces heterotopic ossification after polysystem extremity trauma
Source: Front Immunol. 2023 Dec 5;14:1280884. doi: 10.3389/fimmu.2023.1280884 (PMC10728492; doi:10.3389/fimmu.2023.1280884)
Supplement: Supplementary Figure 2 — Relative expression levels of early signaling genes involved in the development of heterotopic ossification including (A) osteogenesis (B) chondrogenesis (C) angiogenesis (D) adhesion and integrins (E) matrix metalloproteinases and tissue inhibitors of metalloproteinases (F) toll-like receptors (G) transcriptional activators and cytoplasmic protein kinases and (H) apoptosis. For this experiment, muscle tissue was collected from the amputation site at POD-7 from rats treated with FAK2 inhibitor (PF-573228;10 mg/kg/day; 10 mg/kg/day, 6.94 µg/kg/min)), vehicle controls, or in healthy muscle from age-matched controls. Relative expression (2-ΔCt) was calculated using an optimal normalization strategy. One-way ANOVAs for each gene were conducted on ΔCt values to determine treatment effects. Tukey-Kramer post-hoc analyses were utilized to determine the source of the significance. Data represents mean relative expression values ± SEM. * indicates p < 0.05, **indicates p < 0.01, *** indicates p < 0.001 and **** indicates p < 0.0001. [file Image_2.pdf]

Supplemental Figure 2:

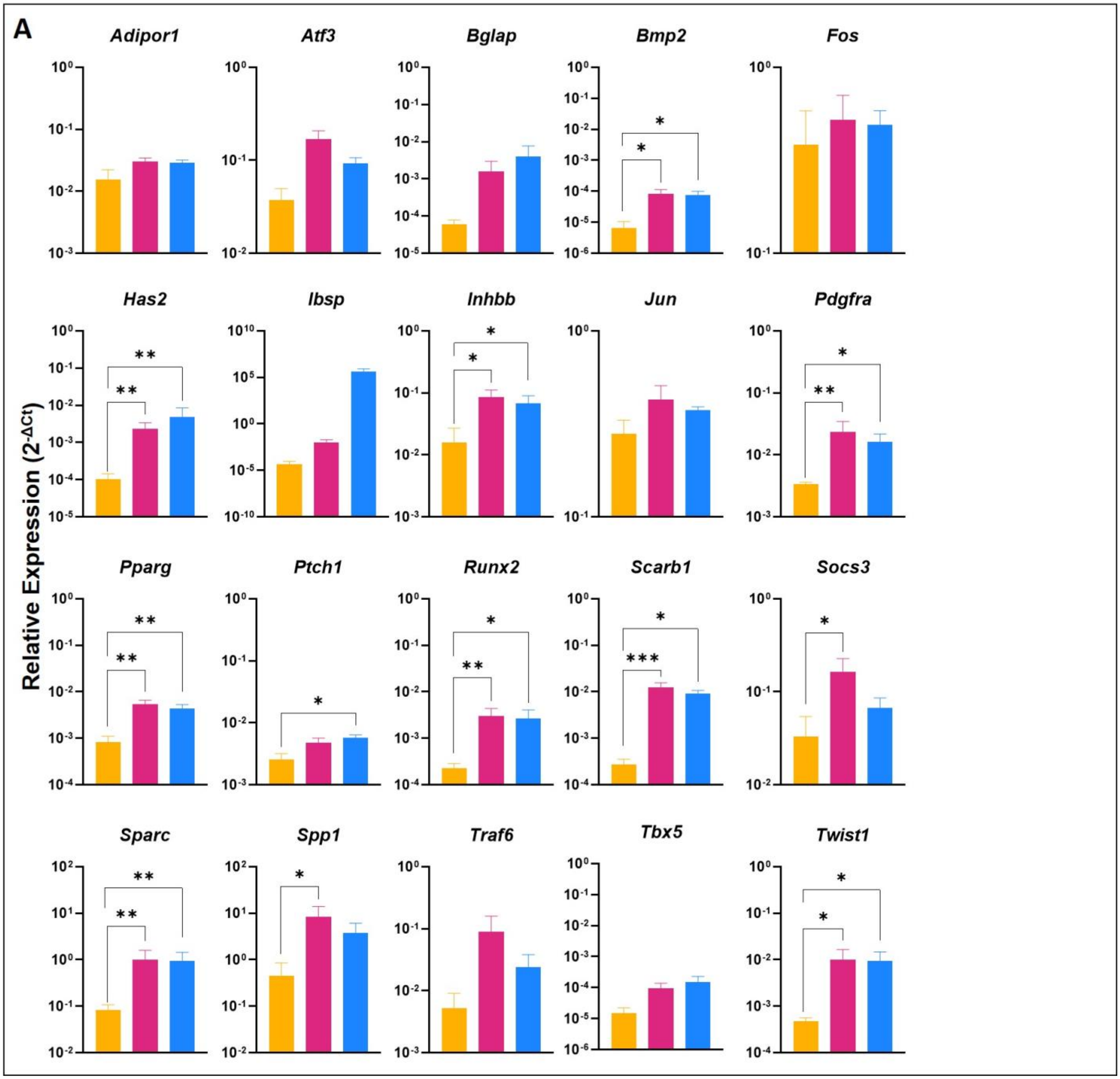

B

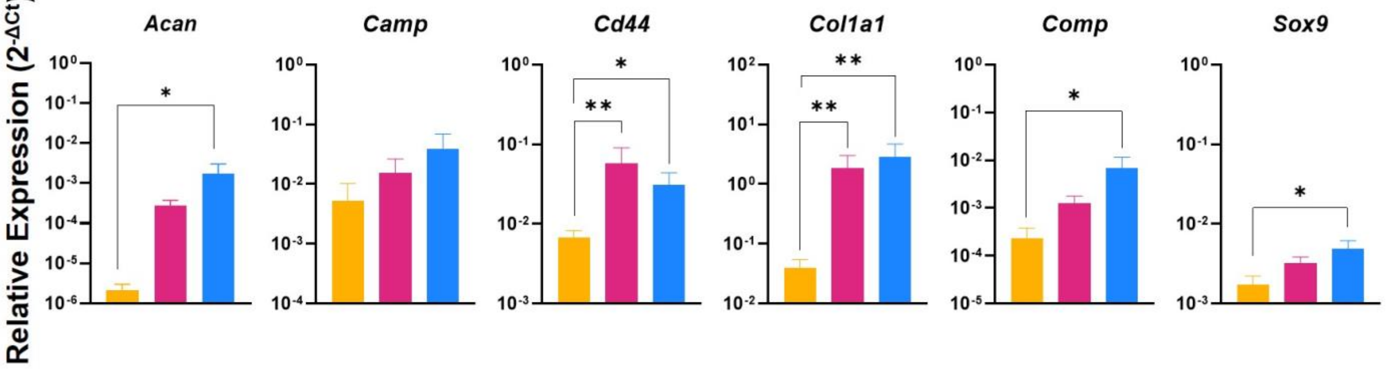
Relative Expression (2-ΔCt)
Acan
Camp
Cd44
Col1a1
Comp
Sox9

C

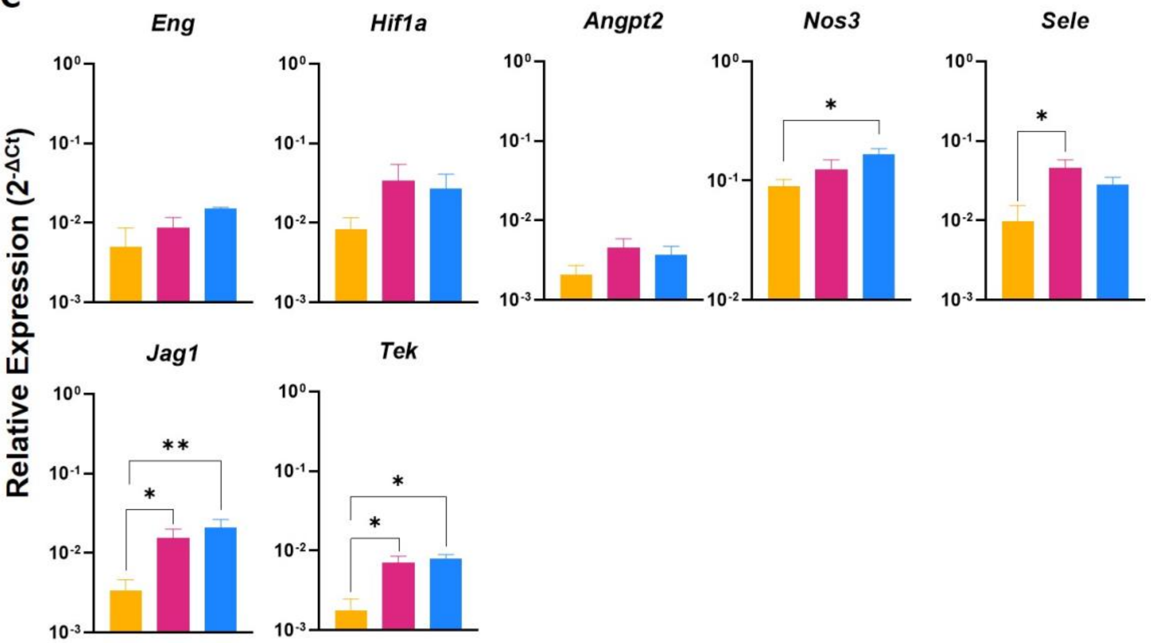
Relative Expression (2-ΔCt)
Eng
Hif1a
Angpt2
Nos3
Sele
Jag1
Tek

D

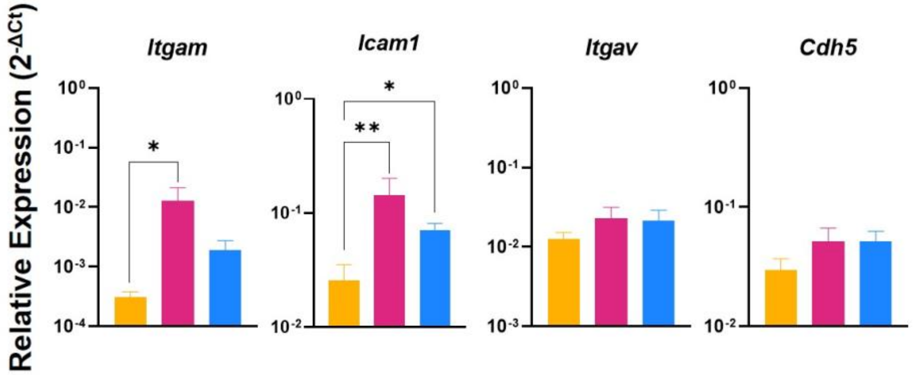
Relative Expression (2-ΔCt)
Itgam
Icam1
Itgav
Cdh5

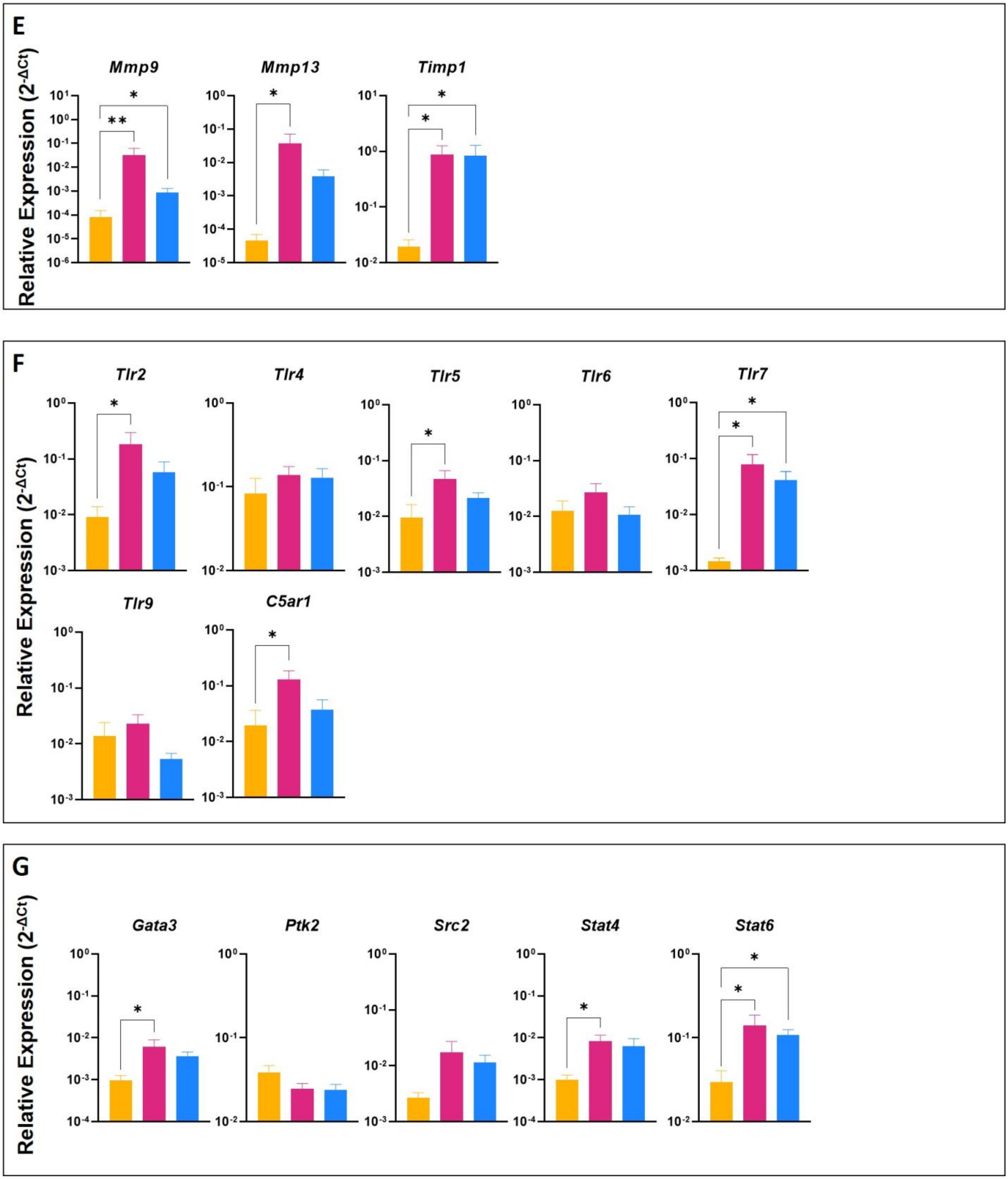
E
Relative Expression (2-ΔCt)
Mmp9
Mmp13
Timp1
**
*
F
Tlr2
Tlr4
Tlr5
Tlr6
Tlr7
Tlr9
C5ar1
G
Gata3
Ptk2
Src2
Stat4
Stat6

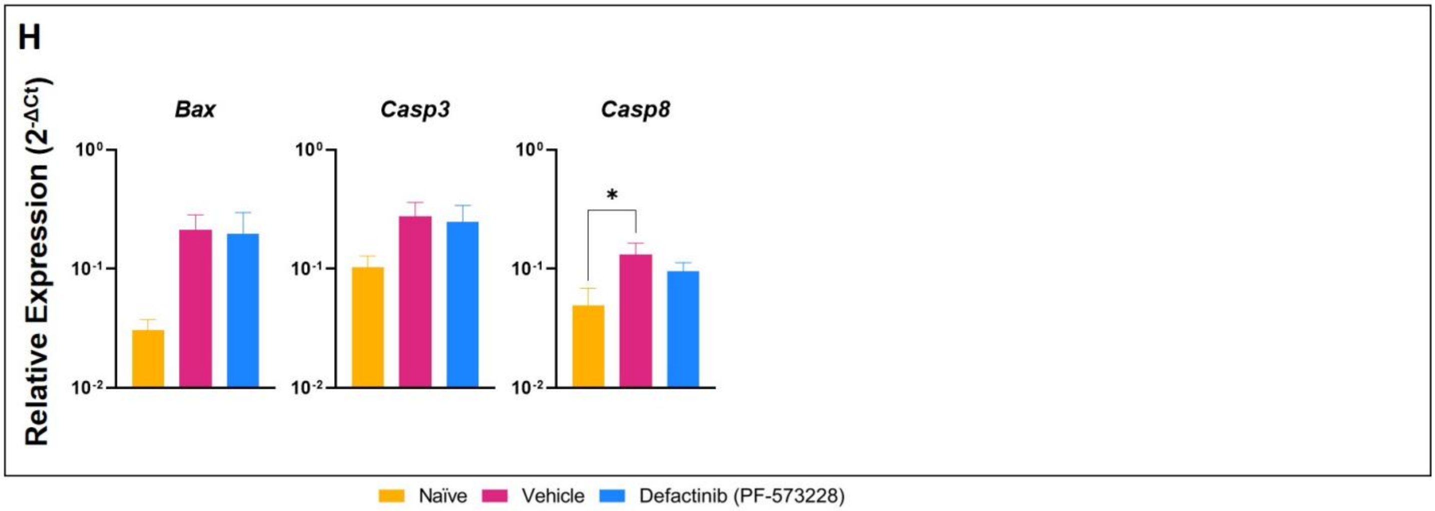
H
Relative Expression ($2^{-\Delta Ct}$)
Bax
Casp3
Casp8
10^0
10^{-1}
10^{-2}
*
Naïve
Vehicle
Defactinib (PF-573228)
