# Supplementary material for: Inhibition of focal adhesion kinase 2 results in a macrophage polarization shift to M2 which attenuates local and systemic inflammation and reduces heterotopic ossification after polysystem extremity trauma
Source: Front Immunol. 2023 Dec 5;14:1280884. doi: 10.3389/fimmu.2023.1280884 (PMC10728492; doi:10.3389/fimmu.2023.1280884)
Supplement: Supplementary file 3 [file Table_1.docx]

**Supplemental Table 1:** Target gene list for the custom arrays for the assessment of osteogenic, chondrongenic, angiogenic, inflammatory, immune cell signaling, and early transcriptional activators in early HO development in injured muscle tissue. This table consists of the target gene name, the gene symbol, RefSeq ID, Bio-Rad assay ID, amplicon length, and the amplicon context sequence provided by Bio-Rad. The gene descriptions were obtained from either Bio-Rad or GeneCards (genecards.org).

| Gene Symbol | Gene Name | Gene Description | Ensembl ID | Bio-Rad Assay ID | Amplicon Length | Efficiency | Amplicon Context Sequence |
| --- | --- | --- | --- | --- | --- | --- | --- |
| *Acan* | Aggrecan core protein precursor | an extracellular matrix proteoglycan; cleaved by ADAMTS1 | ENSRNOG00000028992 | qRnoCID0054298 | 109 | 92 | CAGGAGAAACAGAGTCAACCGCTGCAGACATTGATGAGTGCCTCTCAAGCCCTTGTCTGAATGGAGCCACCTGCGTGGATGCCTTGGACACTTTCACATGCTTATGCCT |
| *Adipor1* | Adiponectin receptor protein 1 | mouse homolog acts as a receptor for adiponectin; facilitates activation of AMP kinase and PPAR-alpha and increased fatty acid oxidation | ENSRNOG00000004143 | qRnoCID0005506 | 106 | 96 | AGAAGATGGAGGAGTTCGTATATAAGGTCTGGGAGGGGCGTTGGAGGGTCATCCCATACGATGTGCTTCCTGACTGGCTGAAAGACAATGACTACCTGCTACATGG |
| *Alpl* | Alkaline phosphatase, tissue-nonspecific isozyme | catalyzes the conversion of an orthophosphoric monoester to an alcohol phosphate | ENSRNOG00000013954 | qRnoCED0002591 | 93 | 100 | AATGGCAAGATCATGGTGTACCCCGAGATCCGTTCCTCGCTGGAGCCCAGATGGGTGGGAAGAGGTGGGGGGCAGTGTCAGCCGTTAATTGAC |
| *Angpt2* | Angiopoietin-2 | Growth factor that is upregulated in multiple inflammatory diseases and is implicated in the direct control of inflammation-related signaling pathways | ENSRNOG00000016696 | qRnoCID0008710 | 101 | 99 | TCTGTATGAGCACTTCTACCTGTCCGGCGAGGAGTCCAACTACAGGATTCACCTTACAGGACTCACAGGCACGGCGGGCAAAATCAGTAGCATCAGCCAAC |
| *Apcs* | Serum amyloid P-component | a component of amyloid P | ENSRNOG00000009086 | qRnoCED0007176 | 80 | 100 | AGTAGCTCATTGTCTCTGCTGTTGACACTGTAGGAGAAAAGACTCTGAGAGCGGGAAAGGTCACTGTAGGCTCGGAAACA |
| *Atf3* | Cyclic AMP-dependent transcription factor ATF-3 | expression is associated with neuronal injury | ENSRNOG00000003745 | qRnoCED0053089 | 85 | 91 | GTTGTTGATGGTGACTGACTCCAGCGCAGAGGACATCCGATGGCAAAGGTGCTTGTTCTGGATGGCGAATCTCAGCTCTTCCTTG |
| *Bax* | Apoptosis regulator BAX | Bcl2-related gene; involved in the regulation of apoptotic cell death | ENSRNOG00000020876 | qRnoCED0002625 | 187 | 97 | GTGGACCTGAGGTTTATTGGCACCTCCCCCAGGCCACCATTCCCACCCCTCCCAATAATTACAAAAGTAAGAAAAATGCCTTTCCCCGTTCCCCATTCATCCCAGGAAAAATGTCATAATTTATGAAGAAAAGACACAGTCCAAGGCAGCAGGAAGCCTCAGCCCATCTTCTTCCAGATGGTGAGTG |
| *Bcl2* | Apoptosis regulator Bcl-2 | an anti-apoptotic protein; involved in inhibiting cell death in many different cell types | ENSRNOG00000002791 | qRnoCED0006419 | 103 | 96 | TGTGACAGCTTATAATGGATGTACTTCATCACGATCTCCCGGTTATCATACCCTGTTCTCCCGGCTTGCGCCATCCTTCCGGGGAAAGAAGCTGCAGGTACCA |
| *Bglap* | Osteocalcin | highly conserved protein associated with mineralized bone matrix; protein is secreted by calcified tissues and is regulated by vitamin D3 | ENSRNOG00000019607 | qRnoCED0007311 | 102 | 103 | GTCGAGTCCTGGAGAGTAGCCAAAGCTGAAGCTGCCGTTGGGCTCCAGGGCAACACATGCCCTAAACGGTGGTGCCATAGATGCGCTTGTAGGCGTCCTGGA |
| *Bmp2* | Bone morphogenetic protein 2 precursor | involved in cellular signaling during limb development; induces bone formation [RGD, Feb 2006] | ENSRNOG00000021276 | qRnoCID0004973 | 79 | 101 | GTGCTTCTTAGACGGACTGCGGTCTCCTAAAGGTCGACCATGGTGGCCGGGACCCGCTGTCTTCTAGTGTTGCTGCTTC |
| *Bmp4* | bone morphogenetic protein 4 precursor | plays a role in induction of cell proliferation | ENSRNOG00000009694 | qRnoCED0008561 | 120 | 101 | GATAATTTTTCAACACCACCTTGTCGTACTCGTCCAGATACAACATGGAAATGGC GCTCAGTTCGGTGGGGACACAGCAGGCCTTAGGGATGCTAGAATTAACGGAGTT GACCAGGGTCTGCACAATGGCATGATTGGTTGAGTTGAGGT |
| *C5ar1* | C5a anaphylatoxin chemotactic receptor | binds complement component 5 and induces Ca+2 release from intracellular stores | ENSRNOG00000047800 | qRnoCED0002472 | 116 | 97 | GAACACGGCCAAGTAGATAATAAGGGCTGCAATGTCCCCAGGTTCCATCTTCGGAATGTAAACGCCATCTGCAGGCATGTCAGGATTTGGGGTTCCATCAGAGTAATCATAGGTGA |
| *Camp* | Cathelicidin antimicrobial peptide precursor | antimicrobial protein that is an integral component of the innate immune system | ENSRNOG00000020733 | qRnoCED0003664 | 109 | 100 | GACTGCTGGTTGAAGTCATCCACAGCACGGAGTACAGCCTCCCTGTAGCTGAGGGTCTGGGAAACGGCTAGAGGCAACCCAAGGCCCAGGAGCAATAGCAGTGACAGTG |
| *Casp3* | Caspase-3 | apoptotic cysteine-aspartic acid protease that may play a role in neuronal cell death regulation and other apoptotic processes | ENSRNOG00000010475 | qRnoCID0006430 | 115 | 100 | CTGCTGTCCAGATATATTCCAGAGTCCATCGACTTGCTTCCATGGATAGTCTTTGTTTCAAAATTATTAATGGATTTTGAATCCACGGAGGTTTCGTTGTTGTCCATGGTCACTT |
| *Casp8* | Caspase-8 | member of the cysteine-aspartic acid protease (caspase) familythat mediates the terminal stage of apoptosis; involved in apoptosis induced by Fas and other stimuli | ENSRNOG00000012331 | qRnoCED0003325 | 61 | 98 | CTGTGCTTGGACCACATCCCGCAGAAGAAGCAGGAGTCCATCAATGATGTCCTGGTGCTAT |
| *Casp9* | Caspase-9 | plays a role in initiation and progression of apoptosis | ENSRNOG00000012944 | qRnoCID0006496 | 66 | 98 | CGGTGGACATTGGTTCTGGCAGAGCTCATGATGTCTGTACTCCAGGGAAGATCGAGAGACATGCAG |
| *Ccl2* | C-C motif chemokine 2 | a monocyte chemoattractant protein | ENSRNOG00000007159 | qRnoCED0009272 | 102 | 94 | GCTAATGCATCCACTCTCTTTTCCACAACCACCTCAAGCACTTCTGTAGAAGTGACCAGTATGACAGAGAACTAGTGTGATTTGGAATGTGATGCCTTAAGT |
| *Ccl3* | C-C motif chemokine 3 | mediates monocyte and neutrophil chemotaxis; may play a role in the pathogenesis of acute lung injury | ENSRNOG00000011205 | qRnoCED0002216 | 112 | 96 | AGGTGGCAGGAATGTTCTGGGGCTCAAGCCCCTGCTCTACACGGGGCCCACGGAGGTTTGGGGGTTCCTTGCTGCCTCTAATCTCAGGCATTTAGTTCCAGCTCAGTGATGT |
| *Ccl12* | Chemokine (C-C motif) ligand 12 precursor | Ligand of CCR2 and induces a strong chemotactic response and mobilization of intracellular calcium ions | ENSRNOG00000029768 | qRnoCED0006489 | 84 | 101 | AGTCACCTGCTGTTATAATGTCGCTAAGCAGAAGATCCACATTCGGAGGCTAAAGAGCTACAGGAAAATCACAAGCAGCCAGTG |
| *Ccr3* | C-C chemokine receptor type 3 | chemokine receptor; may have a role in inflammation and HIV infection | ENSRNOG00000006736 | qRnoCED0055609 | 118 | 92 | GGATTCACTATGTTCTGTGGAATGAGTGGGGTTTTGGCCACTGCATGTGTAAAATGCTCTCTGGGCTTTATTACCTGGCCTTGTACAGCGAGATCTTTTTCATCATCCTGCTGACAAT |
| *Ccr4* | C-C chemokine receptor type 4 | mouse homolog is a chemokine receptor that binds both macrophage inflammatory protein-1 alpha and RANTES | ENSRNOG00000010315 | qRnoCED0005644 | 85 | 99 | ACTTGAGCCTCTTGTACTTGAACAGCACCAGAACCACAACAGAATTCCCAAACAGACCCAACAGAAAGACCAAGGAGTAGAGAGG |
| *Ccr6* | C-C chemokine receptor type 6 | important for B-lineage maturation and antigen-driven B-cell differentiation, and it may regulate the migration and recruitment of dentritic and T cells during inflammatory and immunological responses | ENSRNOG00000012964 | qRnoCED0052490 | 92 | 90 | GTCAGTGGCATGAGTAACTGCCCAGAATGGTAGGGTGAGGACAAAGAGTATGTCTGTGATGGCCATGTTCAATAGGTAGACGTCAGTCATGG |
| *Ccr8* | Protein Ccr8 | Plays a role in regulation of monocyte chemotaxis and thymic cell apoptosis | ENSRNOG00000026759 | qRnoCED0001453 | 113 | 98 | GTATCTGGCCGTCTTATACTGCATCTTGTTTGTGCTGGGCCTTCTGGGAAACAGCCTGGTCATCTTGGTCCTTGTGGCCTGCAAGAAACTGAGGAGTATCACGGACGTCTACC |
| *Cd4* | T-cell surface glycoprotein CD4 | MHC class II binding protein that may be a candidate gene for collagen-induced arthritis; human CD4 is a receptor required for HIV infection | ENSRNOG00000016294 | qRnoCED0002294 | 64 | 101 | AGATGCCACTGTCCTGAATCCTTAGGCTGTGCGTGGAGAAAGCTTTGGAGTCCTTGACAATGTT |
| *Cd8a* | T-cell surface glycoprotein CD8 alpha chain | increased expression on mast cells is induced by nitric oxide; may play a role in inflammatory response | ENSRNOG00000007178 | qRnoCED0001354 | 106 | 94 | GGATGCTCTTGGCTCTTCCGGAACTCCAGCTCCGAACTCCTCCAGCCCACCTTCATCATCTATGTATCTTCATCCCGGAGCAAGCTGAACGATATACTGGATCCGA |
| *Cd14* | Monocyte differentiation antigen CD14 | component of the lipopolysaccharide receptor complex; mediates LPS-induced neuroinflammation and inflammatory response | ENSRNOG00000017819 | qRnoCED0008863 | 82 | 101 | AAGTTGAGTGAGTGTGCTTGGGCAATACTTAGTACCTTGAGTCCAGGCTTTAGCCACTGCTGCAGTTCTGCGAGCCAGGTAT |
| *Cd40* | Tumor necrosis factor receptor superfamily member 5 precursor | may mediate chronic inflammation including arteriosclerosis; interacts with ligand CD154 and plays a crucial role in humoral and cellular immunity, and in T-cell-mediated inflammatory responses | ENSRNOG00000018488 | qRnoCID0003897 | 120 | 91 | CTGATCTCGCTCTGCAATGCTGCCTTTGCCTCAGCTGTGCGCGCTCTGGGGCTGCTTGTTGACAGCGGTCCATCTAGGACAGTGTGTTACGTGCAGTGACAAACAGTACCTCCAAGGTGG |
| *Cd40lg* | CD40 ligand | CD40 ligand | ENSRNOG00000000871 | qRnoCID0009370 | 101 | 99 | TTGCAGCACATGTTGTAAGTGAGGCCAACAGTAATGCAGCATCTGTTCTTCAGTGGGCGAAGAAAGGATATTATACCATGAAAAGCAACTTGGTAGTGCTG |
| *Cd44* | CD44 antigen precursor | adhesion molecule involved in migration and cell fusion in osteoclasts that also plays a role in cellular metastasis | ENSRNOG00000006094 | qRnoCED0004179 | 86 | 101 | GCTCTTCTCAATGGTGGATCCACTGCTGACATCCTCATCTATAATGTTTGAGGCATCGATGTCTTCTTGGTGTGTTCTATACTCGC |
| *Cd80* | T-lymphocyte activation antigen CD80 | cell surface protein that is part of the immune response; may be involved in induction of experimental autoimmune anterior uveitis | ENSRNOG00000001527 | qRnoCID0009234 | 120 | 97 | GTCTCAGGTTCATTCATCTCTTTGTGCTGCTGCTGGTTGGTCTTTTCCAGATATCTTCAGGTATTGTCGGCCAAGTGTCCAAGTCGGTGAGAGAAAAGGCATTGCTGTCCTGTGATTACA |
| *Cd86* | T-lymphocyte activation antigen CD86 precursor | plays a role in T-cell activation and proliferation | ENSRNOG00000038835 | qRnoCED0008398 | 84 | 96 | AACTAATGAGTATGGCGACAACATGCAGATATCACAAGACAATGTCACAAAGCTGTTCAGTGTCTCCATCAGCCTATCTCTTCC |
| *Cdh5* | Cadherin-5 |  | ENSRNOG00000013324 | qRnoCID0007372 | 114 | 100 | TGTCCATACTTGACTGTGATGTTGGCGGTATTGTCGTGGTTGTTGATGAGGGTGAAGTTGCTGTCCTCGTTCTTCAGGGCAAACTTGAACTTTGTGTTTACTGGCACCACGTCC |
| *Ckm* | Creatine kinase M-type | catalyzes regeneration of ATP; expression is induced by p53 | ENSRNOG00000016837 | qRnoCED0005864 | 97 | 101 | CAAGTCGCAGGAGGAGTACCCAGACCTCAGCAAACACAACAACCACATGGCCAAGGTGCTGACTCCTGACCTCTACAATAAGCTTCGAGACAAGGAG |
| *Col1a1* | Collagen alpha-1(I) chain | extracellular matrix collagen protein | ENSRNOG00000003897 | qRnoCED0007857 | 110 | 95 | GAGTATGGAAGCGAAGGTTCCGATCCTGCCGATGTCGCTATCCAGCTGACCTTCCTGCGCCTGATGTCCACCGAGGCCTCCCAGAACATCACCTATCACTGCAAGAACAG |
| *Col2a1* | Collagen alpha-1(II) chain precursor | structural component of the cartilage extracellular matrix | ENSRNOG00000022282 | qRnoCED0005744 | 73 | 100 | TTCGTCCAGGTAGGCAATGCTGTTCTTACAGTGGTAGGTGATGTTCTGGGAGCCCTCAGTGGACAGTAGACGG |
| *Col10a1* | collagen, type X, alpha 1 | extracellular matrix collagen protein; may participate in regulation of Meckel's cartilage development | ENSRNOG00000000545 | qRnoCID0007994 | 120 | 96 | CGTTTTATGCTGAGCGGTACCAAACTCCCACAGGTGTGAAGAGCGCACTTGCCA GCCCCAGGACACAATACTTCATCCCATACCCTATAAAGGGTAAAGAGATTTCAGT AAGAGGAGAACAAGGCATTCCTGGTCCACCAGGCCCAGCTG |
| *Comp* | Cartilage oligomeric matrix protein precursor | extracellular matrix protein that interacts with fibronectin and may play a role in extracellular matrix assembly and molecular interactions | ENSRNOG00000048472 | qRnoCID0001154 | 105 | 94 | GGACAGTTGTCAGCTACATTTCGGATTCGATCGCCATCTATGTCGTCGTCGCAGGCATCGCCCTGGCCATCCCGGTCTGTATCTTTCTGGTCATCATTCTTCTGG |
| *COX2* | Cytochrome c oxidase subunit II (mitochondrion) | this protein plays a role in endothelial adherence junction assembly and maintenance | ENSRNOG00000030371 | qRnoCED0052820 | 91 | 95 | ACCAGGTGAACTTCGTCTATTAGAAGTTGATAATCGGGTAGTCTTACCAATAGAACTTCCAATCCGTATACTAATCTCATCCGAAGACGTC |
| *Crp* | C-reactive protein | glycoprotein of the acute phase response | ENSRNOG00000000053 | qRnoCED0002177 | 101 | 101 | AGGAGCAGGACTCGTATGGCGGTGGCTTTGACGCGAATCAGTCTTTGGTGGGAGACATTGGAGATGTGAACATGTGGGACTTTGTGCTATCTCCAGAACAG |
| *Csf2* | Granulocyte-macrophage colony-stimulating factor | plays a role in alveolar epithelial fluid transport | ENSRNOG00000026805 | qRnoCED0004359 | 92 | 103 | GCAGTTCGTCTGGTAGTGGCTGGCTATCATGGTCAAGGCGCCATTGAGTTTGGTGAGGTTGCCCCGTAGACCCTGCTTGTATAGCTTCAGGC |
| *Csf3* | Granulocyte colony-stimulating factor precursor | putative hematopoetic growth factor for neutrophils; used as a treatment for cyclic hematopoesis in humans and dogs | ENSRNOG00000008525 | qRnoCED0001885 | 117 | 98 | GCACTATGGTCAGGACAAGAGGCCATTCCCCTGCTCACCGTCAGCTCTCTGCCACCATCCCTGCCTTTGCCCCGAAGCTTTCTGCTTAAGTCCTTGGAGCAAGTGAGGAAGATTCAG |
| *Cx3cl1* | Fractalkine precursor | chemokine that binds its receptor CX3CR1; induces chemotaxis and increased intracellular calcium levels in microglia | ENSRNOG00000016326 | qRnoCED0007474 | 80 | 102 | GACAGAACGAGTTGTTGGTGGTGATGGTGGTGATGGCTCTGTACACGGGAGAGAGAGACCGGGATAGATAGCACCGCAGC |
| *Cxcl1* | Growth-regulated alpha protein precursor | acts as a neutrophil chemoattractant; may play a role in acute phase inflammatory response | ENSRNOG00000002802 | qRnoCED0003672 | 84 | 100 | TCACACATTTCCTCACCCTAACACAAAACACGATCCCAGACTCTCATCTCTCCGCCCTTCTTCCCGCTCAACACCTTCTAGCAC |
| *Cxcl2* | C-X-C motif chemokine 2 | chemokine involved in the pulmonary inflammatory response | ENSRNOG00000002792 | qRnoCED0003624 | 102 | 99 | GCTGACTGAACACATTGAACATTATTACAATAAACTTCAACATATTAAAATGACCTCTTAAGATACTACAGTGAGCTGGCCAATGCATATCTTTAAATATCA |
| *Cxcl5* | C-X-C motif chemokine 5 precursor | member of the CXC chemokine family, is a neutrophil chemoattractant and is rapidly induced in response to muscle injury | ENSRNOG00000002843 | qRnoCED0002198 | 72 | 99 | TGAATGACTTTCTTTATCAACGGAGCTTCTGGGTCAAGACAAACATTATCCTTCTGGTTCTTCAACTTAGCT |
| *Cxcl10* | C-X-C motif chemokine 10 | induces DNA synthesis, cell proliferation, and cell migration in vascular smooth muscle cells; may play a role in vascular remodeling | ENSRNOG00000022256 | qRnoCED0009075 | 99 | 97 | TCTAAGAGCTGGTCCGAATCTTCCCTCAGGCAGCTATGACGGCTCTCCTAGCTCTGTTCTGTAAGCTATGTGCAGGTACTAATCTCTTCAGCATGTGCC |
| *Cxcr3* | C-X-C chemokine receptor type 3 | binds interferon-inducible protein-10 and induces intracellular calcium mobilization; increased expression occurs in response to focal stroke | ENSRNOG00000003305 | qRnoCED0006002 | 85 | 98 | ACTAGCCTCATAGCTCGAAAGCGCCTCTGGCCTCTGGAGACCAGCAGCACAGCCAGGATATGGGCATAGCAGTAGGCCATGACTA |
| *Eng* | Endoglin | Vascular endothelium glycoprotein that plays an important role in the regulation of angiogenesis | ENSRNOG00000050190 | qRnoCID0003750 | 198 | 98 | CTACAAGACTGTCTCCATGCGCCTGAACATCGTCAGTCCTGACCTGTCTGGCAAAGGCCTCGTCCTGCCCTCTGTACTGGGCATCACCTTTGGTGCCTTCCTCATTGGGGCCCTGCTCACAGCTGCACTCTGGTACATCTATTCTCACACACGTGAGTATCCCAAGCCTCTCCACCCCCTTCCTACAGTGAGTGCTCA |
| *Faslg* | Tumor necrosis factor ligand superfamily member 6 | ligand that binds the Fas receptor; plays a role in induction of apoptosis | ENSRNOG00000002978 | qRnoCED0003046 | 95 | 103 | TGCCAGTTCCTTCTGTAGATGAAAGAGTTGATACATTCCTAACCCCATTCCAACCAGAGCCACCAGCACCATGAAAAATATCACCGGTAGCCACA |
| *Fos* | Proto-oncogene c-Fos | an immediate early gene encoding a nuclear protein involved in signal transduction | ENSRNOG00000008015 | qRnoCED0002247 | 109 | 99 | AGCTCTCAGGTGTCACTACAAACAATACACTCCATGCGGTTGCTTTTGATTTTTTGTTTTGTTTTTTTTTTGTTTTGTTTTTTTGTTTTTTTGCTACATCTCCGGAAGA |
| *Foxp3* | Forkhead box protein P3 | Transcriptional regulator which is crucial for the development and inhibitory function of regulatory T-cells (Treg) | ENSRNOG00000011702 | qRnoCED0052464 | 109 | 99 | GTAAAGGGTGGTCTTACAGGGGCATAGAGTAGTGGAAAAGGGGGCAATAGCTTGCTGTTATCTCTGAGGTCCCTTTTGTCTTATCAGGATGCTAAGAAGGATGATGCTG |
| *Gata3* | GATA binding protein 3 | transcription factor which is responsible for Th2 commitment during anterior chamber associated immune deviation development | ENSRNOG00000019336 | qRnoCID0008066 | 105 | 93 | ATGTAAGTCGAGGCCCAAGGCACGATCCAGCACAGAAGGCAGGGAGTGTGTGAACTGCGGGGCAACCTCTACCCCACTGTGGCGGCGAGATGGTACTGGGCACTA |
| *Grb2* | Growth factor receptor-bound protein 2 | acts as a link between tyrosine kinase receptors and Ras signaling | ENSRNOG00000003990 | qRnoCED0006592 | 114 | 104 | CTGTGTCCTTTTTTCGCGTTCTTTCTTTCCTGTCCAGTGCATGATGTTTAAGGCCACATATAGTCCAGCTGATGCCAATAATAAAAGAAAAGAAACCAAGTGGGCTGGTATTTTCTCTATGCAAATGTCTGTGGAGATGGATGG |
| *Has2* | Hyaluronan synthase 2 | membrane protein that synthesizes hyaluronan, a high molecular weight polysaccharide that is a component of the extracellular matrix; may be important for epidermal proliferation, thickness and differentiation | ENSRNOG00000004854 | qRnoCID0008940 | 86 | 101 | ACAGTGTGGTTCCGATTATTCTCAGGACACATAGAAACCTCTCACAATGCATCTTGTTCAGCTCCTGCTCATAGACTCGTCCTCCG |
| *Havcr1* | Hepatitis A virus cellular receptor 1 homolog precursor | may be a member of the mucosal addressin cell adhesion molecule family; may be involved in restoration of the morphological integrity and function to postischemic kidney | ENSRNOG00000007243 | qRnoCID0004444 | 85 | 97 | AACACCATGGTTCAACTTCAAGTCTTCATTTCAGGCCTCCTGCTGCTTCTTCCAGGCTCTGTAGATTCTTATGAAGTAGTGAAGG |
| *Hif1a* | Hypoxia-inducible factor 1-alpha | regulates transcription in response to low oxygen; may play a role in vascular biology | ENSRNOG00000008292 | qRnoCID0006918 | 96 | 101 | CTGATGGAAGCACTAGACAAAGCTCACCTGAGCCTAACAGTCCCAGTGAGTACTGCTTTGATGTGGACAGCGATATGGTCAATGTATTCAAGTTGG |
| *Hprt1* | Hypoxanthine-guanine phosphoribosyltransferase | catalyzes the conversion of IMP and diphosphate to hypoxanthine and 5-phospho-alpha-D-ribose 1-diphosphate | ENSRNOG00000031367 | qRnoCED0057020 | 79 | 98 | GTAGATGGCCACAGGACTAGAACGTCTGCTAGTTCTTTACTGGCCACATCAACAGGACTCTTGTAGATTCAACTTGCCG |
| *Hspb1* | Heat shock protein beta-1 | putative heat shock protein; human homolog may suppress polyglutamine-mediated cell death and mutations in human gene are associated with various neuropathies and some forms of Charcot-Marie-Tooth disease | ENSRNOG00000023546 | qRnoCED0001063 | 173 | 93 | GCTGAGTTGCCGGTTGAGCGCCCGGCTGAAGGCGGGCGCGGCCAGGGTCACTGCTGCGGGGCCCTCGGCGGTCGCGGCGGGCAGAGGGCGCACATAGCCGGGCCAACCAGCGGAGCTGAACCACTGAGACCACTCATCGGGAAACCGAGGCACCCCGAAAGCTTGATCGAAGA |
| *Ibsp* | Bone sialoprotein 2 precursor | extracellular matrix protein that binds hydroxyapatite; involved in bone formation | ENSRNOG00000002158 | qRnoCED0005305 | 83 | 99 | CGTTGTTCTCGTCATAGGTTTCATACGCAGTGTTGTACTCGTTGCCTATTTGTTCGTATTCTTCCCCATACTCAACCGTGCTG |
| *Icam1* | Intercellular adhesion molecule 1 | cell adhesion molecule; ligand for leukocyte adhesion molecule LFA-1 | ENSRNOG00000020679 | qRnoCED0005284 | 94 | 100 | GTTGGTGTGTTGGTACTGATCATTGCGGGCTTCGTGATCGTGGCGTCCATTTACACCTATTACCGCCAGAGGAAGATCAGGATATACAAGTTAC |
| *Ifnar1* | Interferon alpha/beta receptor 1 isoform 2 | Binding and activation of the receptor stimulates Janus protein kinases, which in turn phosphorylate several proteins, including STAT1 and STAT2 | ENSRNOG00000028594 | qRnoCED0005522 | 82 | 96 | AACCGCCTAATCCTTGTTCACTGATAGTTCTTGGGGAAGCTGAGCCGTCTAAGAACCGTGTAAAAGACAAGAGTGAGGTGCT |
| *Ifnb1* | Interferon beta | suppresses the growth of rat glioma cells | ENSRNOG00000006268 | qRnoCED0002914 | 99 | 93 | GACATTCTGGAGCATCACTTGAATGGCAAAGGCAGTGTAACTCTTCTCCATCTGTGACGGGTGCATCACCTCCATAGGGATCTTGAAGTCCGTCCTGTA |
| *Ifng* | Interferon gamma | an immune molecule produced by T lymphocytes in response to mitogens or antigens | ENSRNOG00000007468 | qRnoCID0006848 | 120 | 97 | TCACTAACTTCTTCAGCAACAGTAAAGCAAAAAAGGATGCATTCATGAGCATCGCCAAGTTCGAGGTGAACAACCCACAGATCCAGCACAAAGCTGTCAATGAACTCATCAGAGTGATTC |
| *Il1a* | Interleukin-1 alpha precursor | proinflammatory cytokine | ENSRNOG00000004575 | qRnoCID0002952 | 111 | 102 | ATGACCTGGAGGCCATAGCCCATGATTTAGAAGAGACCATCCAACCCAGATCAGCACCTCACAGCTTCCAGAATAATTTGAGATACAAATTGATAAGGATCGTCAAGCAGG |
| *Il1b* | Interleukin-1 beta precursor | an inflammatory cytokine | ENSRNOG00000004649 | qRnoCID0004680 | 120 | 98 | GACAGAACATAAGCCAACAAGTGGTATTCTCCATGAGCTTTGTACAAGGAGAGACAAGCAACGACAAAATCCCTGTGGCCTTGGGCCTCAAGGGGAAGAATCTATACCTGTCCTGTGTGA |
| *Il1r1* | Interleukin-1 receptor type 1 | receptor for interleukin-1; involved in inflammatory response signaling | ENSRNOG00000014504 | qRnoCID0007565 | 82 | 98 | GGACAGACCTGTGATTATGAGCCCACGGAATGAGACGATGGAAGCTGACCCAGGATCCACGATACAACTGATCTGCAACGTC |
| *Il2* | Interleukin-2 | cytokine produced by T-cells in response to antigen or mitogen stimulation | ENSRNOG00000017348 | qRnoCED0006493 | 92 | 97 | AGTCATTGTTGAGATGATGCTTTGACAGATGGCTATCCATCTCCTCAGAAATTCCACCACAGTTGCTGGCTCATCATCGAATTGGCACTCAA |
| *Il4* | Interleukin-4 | Th2-type cytokine; may be involved in inflammatory response in eosinophils | ENSRNOG00000007624 | qRnoCID0002254 | 109 | 98 | GAGCGTGGACTCATTCACGGTGCAGCTTCTCAGTGAGTTCAGACCGCTGACACCTCTACAGAGTTTCCTCAGTTCACCGAGAACCCCAGACTTGTTCTTCAAGCACGGA |
| *Il5* | Interleukin-5 | cytokine with B-cell growth factor activity | ENSRNOG00000008111 | qRnoCED0001585 | 102 | 99 | GGATGCTTCTGTGCTTGAACGTTCTAACTCTCAGCTGTGTCTGGGCCATTGCTATGGAGATCCCCATGAGCACAGTGGTGAAAGAGACCTTGATACAGCTGT |
| *Il6* | Interleukin-6 | A cytokine with a wide variety of biological functions in immunity, tissue regeneration, and metabolism. | ENSRNOG00000010278 | qRnoCID0053166 | 120 | 94 | CAGACCAGTATATACCACTTCACAAGTCGGAGGCTTAATTACATATGTTCTCAGGGAGATCTTGGAAATGAGAAAAGAGTTGTGCAATGGCAATTCTGATTGTATGAACAGCGATGATGC |
| *Il10* | Interleukin-10 | factor involved in the inhibition of cytokine synthesis | ENSRNOG00000004647 | qRnoCID0005930 | 80 | 98 | CCTTCAGTCAAGTGAAGACTTTCTTTCAAAAGAAGGACCAGCTGGACAACATACTGCTGACAGATTCCTTACTGCAGGAC |
| *Il12a* | Interleukin-12 subunit alpha precursor | mouse homolog is a subunit of heteromeric cytokine interleukin 12; loss of function is associated with experimental autoimmune encephalomyelitis | ENSRNOG00000009468 | qRnoCID0006153 | 99 | 98 | CTGCCAAGTGTCTTAACCAGTCCCAAAACCTGCTGAAGACCACGGACGACATGGTGAGGACGGCCAGAGAAAAATTGAAACATTACTCTTGCACTGCTG |
| *Il13* | Interleukin-13 | cytokine; involved in inflammatory and immune responses | ENSRNOG00000007652 | qRnoCID0008414 | 120 | 101 | CATTGCAACTGGAGATGTTGGTCAGGGATTCCAGGGCTGCACAGAACCCGCCAGCTGTCAGGTCCACGCTCCATACCATGCTGCTGTTGCACAGGGAAGTCTTCTGGTCTTGTGTGATGT |
| *Il17ra* | Interleukin 17 receptor A | a proinflammatory cytokine secreted by activated T-lymphocytes | ENSRNOG00000011153 | qRnoCID0003749 | 101 | 93 | GAACTGCAGAGTCGAGAATAGTACTTGCCTGGATGACAGCTGGATTTACCCTCGAAACCTGACGCCGTCTTCCCCGAAAAACATCTATCTCCATCTGAACG |
| *Il23a* | Interleukin-23 subunit alpha | a pro-inflammatory cytokine; high levels of expression may be associated with rheumatoid arthritis, psoriasis, and multiple sclerosis | ENSRNOG00000003254 | qRnoCED0006916 | 91 | 101 | AGTCAGTCCATGTTGGTAGTTCTGTTAGTTCTTAGTGCTGGGTTCTGTTAGAACTGAAGGACTAAGCAGGCAGTTACAGAGCTTCCGTCTG |
| *Inhbb* | Inhibin beta B chain precursor | beta subunit of inhibin; involved in regulation of pituitary FSH secretion | ENSRNOG00000047726 | qRnoCED0054847 | 86 | 99 | TCTGACTGGTCTTCTGTGAAGCGGGAGAACGTTCTGTCAGGCCTCACCTTGGCACTCTTCACAACCCAATGAACAACCACACTCAG |
| *Itgam* | Integrin alpha-M precursor | mouse homolog is an integrin alpha subunit that interacts with integrin beta 2 subunit to form Mac-1; involved in immune response | ENSRNOG00000019728 | qRnoCID0002800 | 120 | 94 | TGCGTGTCAAGAAGAAGTAGTCAAAAACAAGGATGCTGGGGAGGTCAGAGTCTGCCTCCATGTCCGCAAGAACACCAAGGACAGGCTGCGAGAAGGAGACATCCAGAGCACTGTCACTTA |
| *Itgav* | Integrin alpha V | Main function includes cell surface adhesion and signaling | ENSRNOG00000004912 | qRnoCID0002761 | 107 | 96 | GCTGTACATTCTCCATTACGACATTGATGGGCCGATGAACTGTACTGCCGACACAGAGATCAACCCTCTGAGAATTAAGACGCCCGAAAAGAATGACACAGCAGCTG |
| *Jag1* | Protein jagged-1 precursor | ligand responsible for activating Notch1 | ENSRNOG00000007443 | qRnoCID0051429 | 106 | 99 | CCAAGCCACTGTTAAGACAGAGCTCAGCAGAGGAACCAGGAAATCTGTTCTGTTTTTCAGAGGGCGCCTCTGAACTCTGACTTCTGCAACAGCAGCGATAAGTGAG |
| *Jak2* | Tyrosine-protein kinase JAK2 | associated with receptors for erythropoietin, prolactin, growth hormones and interleukin-3; phosphorylated when activated by cytokines | ENSRNOG00000015547 | qRnoCID0004742 | 103 | 102 | GATGAGTCAACCAGGCATAATATACTCTACAGAATAAGGTTTTACTTTCCTCATTGGTACTGCAGTGGCAGCAACAGAACCTATAGGTACGGAGTATCTCGTG |
| *Jun* | Transcription factor AP-1 | transcription factor; acts as a protooncogene | ENSRNOG00000026293 | qRnoCED0006525 | 119 | 101 | ATTCTGGCTATGCAGTTCAGCTAGGGCGCGCACGAAGCCTTCGGCGAAGCCCTCCTGCTCGTCGGTCACGTTCTTGGGGCACAAGAACTGAGTGGGGGTCGGTGTAGTGGTGATGTGCC |
| *Kdr* | Vascular endothelial growth factor receptor 2 precursor | receptor for vascular endothelial growth factor (VEGF) | ENSRNOG00000046829 | qRnoCID0004950 | 119 | 104 | CTATGTCTGCTCTGCTCAAGACAAGAAGACCAAGAAAAGACATTGCCTAGTCAAGCAGCTCGTCATCCTAGAGCGCATGGCACCCATGATCACTGGAAATCTGGAGAATCAGACAACAA |
| *Lbp* | Lipopolysaccharide-binding protein precursor | binds lipopolysaccharide on outer membrane of gram-negative bacteria; involved in immune response | ENSRNOG00000014532 | qRnoCID0007468 | 80 | 97 | TGTCGATATCCGCTGTGACTGGCAGAGTTTGGAGATAAGGCTGCAGATCAGAGGTCACAGACTTCTGGATCATCTCACAA |
| *Lcn2* | Neutrophil gelatinase-associated lipocalin | plays a role in IL3 withdrawal-induced apoptosis | ENSRNOG00000013973 | qRnoCED0001706 | 68 | 99 | CTGACGAGGATGGAAGTGACGTTGTAGCTATTGTCTTCCTGTAGCTCATAGATGGTGCTGTACATGGT |
| *Lyz2* | Lysozyme C-1 precursor | widely expressed; may mediate analgesic, anti-inflammatory, hemostatic, anti-allergy, anticancer, and antibiotic functions | ENSRNOG00000005825 | qRnoCID0051084 | 197 | 88 | GGGATCCCTCACAACTCTCTTCGCACATTGTATGGCTTGAGTGATGTCATCCTGCAGCAGAGCGCTGCAGGGTATCCCACAGGCGTTCTTTGCTCTTGGGGTTTTGCCGTCATTACACCAGTATCGGCTATTGATCTGAAATATCCCATAGTCGGTGCTTTGGTCTCCAGGGTTGTAGTTTCTGGCTTGTGTGTTAT |
| *MBL2* | Mannose Binding Lectin 2 | Binding of this protein activates the classical complement pathway | ENSRNOG00000011637 | qRnoCID0017891 | 116 | 96 | TCCATACGAACAAAGCTTGATCCATGCAGCAACAGAGTCTAAAGCAAACAGCGAAAACCAGCATTATACTCTAGAAAAGCAAAGCGTTCACATTTCAGTTGTGTCTGTTGAGTTCC |
| *Mmp13* | collagenase 3 precursor | enzyme that degrades collagen type I | ENSRNOG00000008478 | qRnoCID0006237 | 87 | 99 | ACTGGCAAAAGCCATTTCATGCTCCCAGATGATGACGTTCAAGGAATCCAGTCTC TCTATGGTCCAGGAGATGAAGACCCCAACCCTAAGCACCCCAAAACACCAGAGA AGTGTGAC |
| *Mmp9* | Matrix metalloproteinase-9 precursor | metalloproteinase involved in extracellular matrix remodeling and bone resorption | ENSRNOG00000017539 | qRnoCED0001183 | 70 | 95 | GGTCGCTCGGATGGTTATCGCTGGTGCGCCACCACCGCCAACTATGACCAGGATAAGCTGTATGGCTTCT |
| *Mpo* | Myeloperoxidase precursor | myeloperoxidase family member that may contribute to hyperoxia-mediated lung injury via nitration of proteins, resulting in reactive nitrogen species | ENSRNOG00000008310 | qRnoCED0008317 | 69 | 98 | TGTTCTTAGACACGGTAGTGATGCCAGTGTTGTCACAGATGATGCGGGGCAAGGAGATGGTAGCCAAGG |
| *Myd88* | Myeloid differentiation primary response protein MyD88 | an adaptor protein which binds toll-like receptors and links them to other downstream signalling molecules | ENSRNOG00000013634 | qRnoCED0002859 | 69 | 100 | GCAGACATGGCAAGCAACCCTGGGCCCCGGTTCTGTACGGTGCGCTCGCTTGTTGAGCTTCCTACTTCC |
| *Nod2* | Nucleotide-binding oligomerization domain-containing protein 2 | primarily expressed in the peripheral blood leukocytes; plays a role in the immune response to intracellular bacterial lipopolysaccharides (LPS) by recognizing the muramyl dipeptide (MDP) derived from them and activating the NFKB protein | ENSRNOG00000014124 | qRnoCID0005178 | 94 | 105 | AAGCCAGCAACATAGTAACCGAGGGATCTTCTTGAACTCATCTCCCATGCTTGGAGTCAGAGCTCCTCTAGTGACTTGTTCTTCTCCAGCATCA |
| *Nos3* | Nitric oxide synthase, endothelial | enzyme that synthesizes Nitric oxide from L-arginine | ENSRNOG00000009348 | qRnoCID0005021 | 117 | 103 | CCACAGTGATGAGGTTGTCCGGGTGTCTAGATCCATGCAGACAGCCACATCCTCAAGTATGTTGTATCGGTGAGGGTCACACAGGTCCCTCATGCCAATCTCTGAACTCATGTACCA |
| *Pdgfra* | Platelet-derived growth factor receptor alpha precursor | acts as a receptor tyrosine kinase for PDGF; may play a role in glial cell generation | ENSRNOG00000002244 | qRnoCID0003041 | 96 | 102 | GTATAATGGCCGCTGTCTTCTTCCTTAGCCCGGATCAGCTTTAATTTGCTTTGATACCTCGTCTCCTGGCTCCTCTGCACATCGGTGGTGATCTCA |
| *Pgk1* | Phosphoglycerate kinase 1 | kinase enzyme that is important for phosphoprotein glycolysis | ENSRNOG00000002467 | qRnoCED0002588 | 78 | 98 | GCTCTCTTCGCTGTATGTAGCCTCTGGTTAGCTTTGTCACTGTTCATGACAGCATGGAAATAACGGTGAGATTCCAGC |
| *Pparg* | Peroxisome proliferator-activated receptor gamma | ligand-activated transcription factor; mediates expression of genes involved in lipid metabolism | ENSRNOG00000008839 | qRnoCID0006036 | 133 | 102 | AGCCTAAGTTTGAGTTTGCTGTGAAGTTCAATGCACTGGAATTAGATGACAGTGACTTGGCCATATTTATAGCTGTCATTATTCTCAGTGGAGACCGCCCAGGCTTGCTGAACGTGAAGCCCATCGAGGACAT |
| *Ppia* | Peptidyl-prolyl cis-trans isomerase A | may play a role in protein folding or intracellular protein transport | ENSRNOG00000006423 | qRnoCID0056995 | 106 | 102 | GCAGACATGGTCAACCCCACCGTGTTCTTCGACATCACGGCTGATGGCGAGCCCTTGGGTCGCGTCTGCTTCGAGCTGTTTGCAGACAAAGTTCCAAAGACAGCAG |
| *Ptch1* | Protein patched homolog 1 | human homolog acts as a receptor for sonic hedgehog | ENSRNOG00000019354 | qRnoCED0051189 | 108 | 93 | GACATTAGCGCCTTCTTCTTTTGGAGTCTGAATCATGAGTTGAGGATTAAACATAGCCTCTTCTCCTATCTTCTGGCGGGTATAATTTAATTCTCGACTCACTCGTCC |
| *Ptk2* | Focal adhesion kinase 1 | tyrosine kinase that is enriched in focal adhesions; may regulate mechanical signal transduction in cardiac myocytes | ENSRNOG00000007916 | qRnoCID0008255 | 117 | 99 | ATAAGATCAAAGATGCTAGATCCAAGCTGTATTTCCTTACTTGCAAAAATGACCTC AGCTCTCCAAGTGTGCACAACTCCATGATTATCCAAACTGGGTTCTCTGTAATGA CTCCAATCAGCTTCACGATGTGAGGATGGTCAAACT |
| *Rag1* | V(D)J recombination-activating protein 1 | expressed in intestinal intraepithelial T lymphocytes | ENSRNOG00000004630 | qRnoCED0004854 | 95 | 107 | TCTTCCTCCATGTCCATCAAAGCAGACACCAAAGCAGAGTCATAGCGGAACCTCTTTGCAATTGTGTCCACTGGGTATTCATCTACGGAGGAGGC |
| *Rplp0* | 60S acidic ribosomal protein P0 | member of the ribosomal protein family; has similarity to other P ribosomal proteins over the carboxyl terminal sequence | ENSRNOG00000001148 | qRnoCED0005242 | 97 | 100 | TAAGCAGGCTGACTTGGTGTGAGGGGCTTAGTCGAAGAGACCGAATCCCATGTCCTCATCGGATTCCTCCGACTCTTCCTTTGCTTCGACCTTGGCT |
| *RPLP2* | Ribosomal Protein Lateral Stalk Subunit P2 | plays an important role in the elongation step of protein synthesis | ENSRNOG00000037607 | qRnoCED0015635 | 60 | 89 | CGCTACGTTGCCTCTTATCTGCTGGCCGCCCTCGGGGGCAACTCCAATCCCAGCGCCAAA |
| *Runx2* | Runt-related transcription factor 2 | essential for osteoblastic differentiation and skeletal morphogenesis and acts as a scaffold for nucleic acids and regulatory factors involved in skeletal gene expression | ENSRNOG00000020193 | qRnoCED0009315 | 74 | 98 | GACACGTACAGCCTAGGAACACAGATGCATTAGGAGTCTGCACCCCCCCCCCAGCCTTACCACACAGCCATTCA |
| *S100b* | Protein S100-B | binds GTPase activating protein IQGAP1; may play a role in cell membrane rearrangement | ENSRNOG00000001295 | qRnoCED0002640 | 91 | 97 | AGACGTCTACTGAGCAGAATTTTGATTCTCGGTCGTGAGTTAGGATGCTAATACTTAGTGTGCGAGATCTAATCGTTCCACCAGTTAGAAC |
| *Scarb1* | Scavenger receptor class B member 1 | plasma membrane receptor for high density lipoprotein cholesterol (HDL) | ENSRNOG00000000981 | qRnoCED0052271 | 174 | 90 | CTTTCTCCGTCTTTCTCCAGCAGGCTGAAAGCCTTAGTTCCCACAGAGGACACAGATCTGTCACTCCTAGGGGCTGGGCAAACATCGGCCTCTTGCGTCACGGCTGGACAAGCAGTTCCAGATCCTGACCAGTCTGCACACACACTCTGGCGCTGTATCTGGCCCTTCTCCTAC |
| *Sele* | E-selectin | facilitates recruitment of leukocytes into sites of inflammation; plays a role in cell adhesion | ENSRNOG00000002723 | qRnoCED0003049 | 78 | 101 | TGATGAAGCAAGTGCGTATTGTCAACGGGACTACACACATCTGGTGGCGATTCAGAACAAGGAAGAGATCAATTACCT |
| *Slc11a1* | Natural resistance-associated macrophage protein 1 | putative metal ion transporter; mouse homolog is a macrophage protein associated with resistance or susceptibility to intracellular pathogens | ENSRNOG00000014956 | qRnoCED0006416 | 110 | 99 | TCTAGAGAGGTAGACAGAACTCGCCGGGGGGATGTTCGAGAAGCCAACATGTACTTCCTGACTGAGGCCACCATCGCCCTCTTCGTGTCATTCATCATCAACCTCTTCGT |
| *Socs3* | Suppressor of cytokine signaling 3 | regulates cytokine levels to modulate inflammation | ENSRNOG00000002946 | qRnoCED0005906 | 110 | 99 | GTTGACAGTCTTCCGACAAAGATGCTGGAGGGTAGCCACGTTGGAGGAGAGAGGTCGGCTCAGTACCAGCGGGATCTTCTCGCCCCCAGAATAGATGTAGTAAGCTCTCT |
| *Sox5l1* | SRY (sex determining region Y)-box 5 | involved in notochord extracellular matrix sheath formation and notochord cell survival | ENSRNOG00000027869 | qRnoCID0008523 | 118 | 98 | ACTCATGTTGAAATCCATCATTCCATGGCTAAATTTCCCTTCTTCATTCTGTTTAACTGCCAACTGCTGAGTCAGGCTCTCCAGTGTTGTTTTTTCCTTTTCTGTCCGGCA GTTGCTGATACCTATGCTGTTCACTACGGCCACCTTC |
| *Sox6* | transcription factor SOX-6 | a transcriptional activator that is required for normal development of the central nervous system, chondrogenesis and maintenance of cardiac and skeletal muscle cells | ENSRNOG00000020514 | qRnoCID0007506 | 84 | 99 | AAAAAATGGAAAGACTAAATACGAGTGAACTTCTTGGAGAAATCAAAGGTACACC TGAGAGCCTTGCAGAGAAAGAACGGCAACTCTCCACCATGATTACCCAGCTGAT CAGCT |
| *Sox9* | Protein LOC100361122 | transcription factor; involved in chondrocyte differentiation and cartilage formation [RGD, Feb 2006] | ENSRNOG00000002607 | qRnoCED0001168 | 91 | 99 | CGCAACAGATCTCCTACAGCCCCTTCAACCTTCCGCACTACAACCCCTCCTACCCAACCATCACGCGCTCGCAGTATGACTACACCGACCA |
| *Sp7* | Transcription factor Sp7 isoform 2 | may act as a transcription factor; mouse homolog is required for bone formation and osteoblast differentiation [RGD, Feb 2006] | ENSRNOG00000014082 | qRnoCED0008486 | 71 | 102 | CCTGGTTGCAAGAGGTGGGGTGCTGGATAGGGAGCTGGGTTAAGGGGGGCAAAGTCAGACGGGTAAGTAGG |
| *Sparc* | SPARC | secreted structural protein; may be a component of collagen and the extracellular matrix [RGD, Feb 2006] | ENSRNOG00000012840 | qRnoCED0004163 | 80 | 105 | TCATCTCTCTCGTACAAGGTGACCAGGACGTTTTTGAGCCAGTCACGCATGCGCAGAGGGAATTCGGTCAGCTCAGAATC |
| *Spp1* | Osteopontin | sialoprotein that binds osteosarcoma cells; may play a role in osteoclast cell function [RGD, Feb 2006] | ENSRNOG00000043451 | qRnoCED0009101 | 64 | 99 | GATTCATCGGAATGGTGAGATTCGTCAGATTCATCCGAGTTCACAGAATCCTCGCTCTCTGCAT |
| *Src* | proto-oncogene tyrosine-protein kinase Src | mediates growth effects of both the precursor and fully processed forms of gastrins on rat intestinal epithelial cells | ENSRNOG00000009495 | qRnoCID0009252 | 96 | 99 | AGCCTGGATGGAGTCGGAGGGCGCCACATAGTTACTGGGGATGTAACCGGTCT GTCCGGTGCTCAGCGAGTGTGCCAGCCACCAGTCTCCCTCTGTGTTATTGACAA TCTGCAGCCGCTCCCCTTT |
| *Stat4* | Signal transducer and activator of transcription 4 | Transcriptional regulator mainly expressed in hematopoietic cells that plays a critical role in cellular growth, differentiation and immune response | ENSRNOG00000050942 | qRnoCID0001087 | 117 | 93 | TCCAGTTGCTGTCTAAGTTGGAAAAGACTCTCTGCCAGTAGGGTAAAGCAGTTCTGAAGCTGGTCCAACCCGTTGTGGAGCGGGCCACCAATGCAGGCGATCTGTTGCCGCTTCTTC |
| *Stat6* | Signal transducer and transcription activator 6 | Carries out a dual function: signal transduction and activation of transcription. Involved in IL4/interleukin-4- and IL3/interleukin-3-mediated signaling | ENSRNOG00000025023 | qRnoCED0001198 | 107 | 94 | GACTGCTACCAGAACACTTCCTGTTCCTGGCCCAGAAGATCTTCAATGACAACAGCCTTAGCATAGAGGCCTTTCAGCACCGCTGTGTGTCTTGGTCACAGTTCAAC |
| *Tbp* | TATA-box-binding protein | binds to TATA box promoter element; involved in activation of eukaryotic genes | ENSRNOG00000001489 | qRnoCID0057007 | 107 | 95 | TCCTTCACCAATGACTCCTATGACCCCTATCACTCCTGCCACACCAGCCTCTGAGAGCTCTGGGATTGTACCACAGCTCCAAAATATTGTATCCACCGTGAATCTTG |
| *Tbx5* | T-box transcription factor TBX5 | T-box genes encode transcription factors involved in the regulation of developmental processes | ENSRNOG00000001399 | qRnoCID0006246 | 73 | 98 | TACCACTGTACCAAGAGGAAAGATGAAGAATGTTCCAGCACCGAGCACCCCTATAAGAAGCCGTACATGGAGA |
| *Tek* | TEK tyrosine kinase, endothelial | mediates development of embryonic vasculature; plays a role in tumors and skin wounds | ENSRNOG00000008587 | qRnoCID0003531 | 76 | 98 | GACACCATCCGAACATCATTAATCTCTTGGGAGCATGTGAACACAGAGGCTACTTATACCTGGCTATTGAGTATGC |
| *Tfrc* | Transferrin receptor protein 1 | receptor for transferrin; involved in regulating stellate cell activation | ENSRNOG00000001766 | qRnoCID0003700 | 66 | 96 | AGCCTCACGAGGAGTATATGTATTCTGGCTCAGCTGCTTGATGATGTCAGTGAACTCTATGGAATT |
| *Tgfb1* | Transforming growth factor beta-1 Latency-associated peptide | binds the TGFbeta receptor; plays a role in regulation of cell growth and proliferation; induces synthesis of extracellular matrix proteins and may play a role in fibrosis | ENSRNOG00000020652 | qRnoCID0009191 | 109 | 99 | GACGTCACTGGAGTTGTCCGGCAGTGGCTGAACCAAGGAGACGGAATACAGGGCTTTCGCTTCAGTGCTCACTGCTCTTGTGACAGCAAAGATAATGTACTCCACGTGG |
| *Tgfb3* | Transforming growth factor beta-3 | involved in epithelial and endothelial cell proliferation and differentiation during development | ENSRNOG00000009867 | qRnoCED0005388 | 101 | 96 | AATCCATGTTCTCACCAGTTTGCCTTCCCCTAACCAACCCACACTTTCTTTACCACAGTGATTCTCAGAGCTAGCAGAAAGAAATGTTCCAGAAGGAAGCC |
| *Timp1* | Metalloproteinase inhibitor 1 | acts as an inhibitor of metalloprotease activity; may play a role in vascular tissue remodeling | ENSRNOG00000010208 | qRnoCID0004258 | 119 | 103 | CGTCGAATCCTTTGAGCATCTTAGTCATCTTGATCTCATAACGCTGGTATAAGGTGGTCTCGATGATTTCTGGGGAACCCATGAATTTAGCCCTTATAACCAGGTCCGAGTTGCAGAAA |
| *Tlr2* | Toll-like receptor 2 precursor | involved in the response to bacteria and injury | ENSRNOG00000009822 | qRnoCED0003882 | 93 | 91 | GTCAGGTGATAGATGTCTCTAATTGACTGTAGACTTCGGGACTCATAGTTGCCGAGACTTAATACCTGAATTTCAAGTTCGTTGAGAGAGGTC |
| *Tlr4* | Toll-like receptor 4 precursor | receptor that functions as the major upstream sensor for hemorrhagic shock and lipopolysaccharide | ENSRNOG00000010522 | qRnoCED0002945 | 117 | 102 | GGTCTAGAAGAGCTGGAATACCTGGACTTTCAGCACTCCACTTTAAAAAAGGTCACAGAATTCTCAGTGTTCTTATCTCTTGAAAAACTTCTTTACCTTGACATCTCTTACACTAAT |
| *Tlr5* | Toll-like receptor 5 | Pattern recognition receptor (PRR) located on the cell surface that participates in the activation of innate immunity and inflammatory response | ENSRNOG00000022067 | qRnoCED0006599 | 111 | 102 | GAGTTCGGTGCTACAACTGGACCTTTCACACGGCTATATCTTCTCCTTGAACCCCCGACTGTTTGAGACGCTGAAGGATTTGAAGAAGCTGAACCTTGCCTTCAACAAGAT |
| *Tlr6* | Toll-like receptor 6 | plays a role in pathogen recognition and activation of the immune response; interacts with Tlr2 | ENSRNOG00000002161 | qRnoCED0009346 | 119 | 97 | CGATAACTGAGAGCATAAGCAGGGAGACATTTATTTACGTGGAGACGGTGTTGAAGTCACTGAAGATAGAGCATGTCACAAACCAAGTGTTCCTCTTTGTGAAGGATGCACTATATTCT |
| *Tlr7* | Toll-like receptor 7 | endosomal receptor that plays a key role in innate and adaptive immunity | ENSRNOG00000004249 | qRnoCED0006963 | 103 | 99 | GAATCTGCTCAACTTAGAGGAATTAGATATCTCCAGAAATTCCCTGAATTCCGTGCCTCCTGGAGTTTTTGAGGGTATGCCACCGAATCTAACGACTCTCTCC |
| *Tlr9* | Toll-like receptor 9 precursor | plays a fundamental role in pathogen recognition and activation of innate immunity | ENSRNOG00000048161 | qRnoCED0009119 | 67 | 99 | CTCAGCCATAACATCCTCAAGACTGTGGATCGCTCCTGGTTTGGGCCCATTGTGATGAACCTGACGG |
| *Tnf* | Tumor necrosis factor Tumor necrosis factor, membrane form Intracellular domain 1 | acts as a cytokine; binds TNF receptors; plays a role in regulation of cell proliferation, induction of apoptosis, and inflammatory response | ENSRNOG00000000837 | qRnoCED0009117 | 112 | 98 | TGGAGTCATTGCTCTGTGAGGCGACTGGCGTGTTCATCCGTTCTCTACCCAGCCCCTGTCCCCGACTCTGACCCCCATTACTCTGACCCCTTTATCGTCTACTCCTCAGAGC |
| *Traf6* | TNF receptor-associated factor 6 | a signal transducer in the NF-kappaB pathway that activates IkappaB kinase (IKK) in response to proinflammatory cytokines | ENSRNOG00000004639 | qRnoCED0005912 | 117 | 99 | TGTAAGACTGTGGTCATGTGGTTGCCAACTGTTCAGTGTGACTGTCATGTAACCTTTCTTGTCTGTTCAGTATAGCTTGGTTTCCACAGCCTGTCGCACATCTTCTGTTGCTTGCAA |
| *Trem1* | Triggering receptor expressed on myeloid cells 1 | amplifies neutrophil and monocyte-mediated inflammatory responses triggered by bacterial and fungal infections by stimulating release of pro-inflammatory chemokines and cytokines, as well as increased surface expression of cell activation markers | ENSRNOG00000022859 | qRnoCID0008319 | 90 | 104 | ACAACATTGTATGTGGAGACACTCGTAGGATCTGTCCCATTGTTGATGGTGACTCCAGGATCAGGAGAGGAAACAACAGCAGTGGACTTG |
| *Twist1* | Twist gene homolog 1 | beta helix loop helix transcription factor | ENSRNOG00000011101 | qRnoCED0001288 | 97 | 98 | GAGACTCTGGAGCTGGATAACTAAAAATAAATCTATATGACAAAGATTTTCTTGGAAATTAGAAGAGCAGAGACCAAATTTCACAAGAATCAGGGCG |
| *Vegfa* | Vascular endothelial growth factor A | mitogen that specifically acts on endothelial cells and has various effects, including mediating increased vascular permeability, inducing angiogenesis, vasculogenesis, endothelial cell growth, promoting cell migration, and inhibiting apoptosis. | ENSRNOG00000019598 | qRnoCED0002159 | 120 | 99 | AGACACAGTGGTGGAAGAAGAGGCCTGGTAATGGCTCCTCCTCCTCCTCCTGGGAACCCCTCGTCCTCTCCCTACCCCACTTCCTGGGTATAGCTCAGGAGGACCTTGTGTGATCAGACC |

**Supplemental Table 2:** Target gene list for the custom cell surface marker arrays. This table consists of the target gene name, the gene symbol, RefSeq ID, Bio-Rad assay ID, amplicon length, and the amplicon context sequence provided by Bio-Rad. The gene descriptions were obtained from either Bio-Rad or GeneCards (genecards.org).

| Gene Symbol | Gene Name | Gene Description | Ensembl ID | Bio-Rad Assay ID | Amplicon Length | Efficiency | Amplicon Context Sequence |
| --- | --- | --- | --- | --- | --- | --- | --- |
| *Arg1* | Arginase-1 | catalyzes the hydrolysis of arginine to ornithine and urea in arginine metabolism; regulates nitric oxide production | ENSRNOG00000013304 | qRnoCID0006520 | 68 | 103 | CTGGTGGAGAAGCTTAAAGAAACAGAGTACAATGTGAGAGACCACGGGGATCTGGCCTTTGTGGATGT |
| *Ccl2* | C-C motif chemokine 2 | a monocyte chemoattractant protein | ENSRNOG00000007159 | qRnoCED0009272 | 102 | 94 | GCTAATGCATCCACTCTCTTTTCCACAACCACCTCAAGCACTTCTGTAGAAGTGACCAGTATGACAGAGAACTAGTGTGATTTGGAATGTGATGCCTTAAGT |
| *Ccr7* | C-C chemokine receptor type 7 precursor | chemokine receptor; involved in the immune response to viruses | ENSRNOG00000010665 | qRnoCID0007211 | 118 | 99 | GACCTCATCTTGGCAGAAGCACACCTGGAAAATGACCAGGAGAGCCACCACCAGCACGTTTTTCGTGGGCTTCCCCAGGTCCATGATGCTCTCTGGGCAGTTGAAGTCACACAGGAAG |
| *Cd14* | Monocyte differentiation antigen CD14 | component of the lipopolysaccharide receptor complex; mediates LPS-induced neuroinflammation and inflammatory response | ENSRNOG00000017819 | qRnoCED0008863 | 82 | 101 | AAGTTGAGTGAGTGTGCTTGGGCAATACTTAGTACCTTGAGTCCAGGCTTTAGCCACTGCTGCAGTTCTGCGAGCCAGGTAT |
| *Cd163* | Scavenger receptor cysteine-rich type 1 protein M130 | Acute phase-regulated receptor involved in clearance and endocytosis of hemoglobin/haptoglobin complexes by macrophages | ENSRNOG00000010253 | qRnoCID0008321 | 107 | 101 | AGAAGATGCTTCTGTGAAGTGCCTCCCAAGAATGACTTTAGAATCACAGCATGGCACAGGTCATTCAACCCTCACTGCACTCCTGGTTTGTGGAGCCATTCTATTGG |
| *Cd3g* | T-cell surface glycoprotein CD3 gamma chain precursor | Part of the TCR-CD3 complex present on T-lymphocyte cell surface that plays an essential role in adaptive immune response. | ENSRNOG00000015945 | qRnoCID0003408 | 109 | 97 | CCTTGGAGACGGCTGTACTGTTCATATTCCCGGTCCTTGAGGGGCTGGTAGACCTGTTCATTTTGCAACAGAGTCTGCTTGTCTGAAGCTCTTGACTGGCGAACTCCAT |
| *Cd4* | T-cell surface glycoprotein CD4 | MHC class II binding protein that may be a candidate gene for collagen-induced arthritis; human CD4 is a receptor required for HIV infection | ENSRNOG00000016294 | qRnoCED0002294 | 64 | 101 | AGATGCCACTGTCCTGAATCCTTAGGCTGTGCGTGGAGAAAGCTTTGGAGTCCTTGACAATGTT |
| *Cd40* | Tumor necrosis factor receptor superfamily member 5 precursor | may mediate chronic inflammation including arteriosclerosis; interacts with ligand CD154 and plays a crucial role in humoral and cellular immunity, and in T-cell-mediated inflammatory responses | ENSRNOG00000018488 | qRnoCID0003897 | 120 | 91 | CTGATCTCGCTCTGCAATGCTGCCTTTGCCTCAGCTGTGCGCGCTCTGGGGCTGCTTGTTGACAGCGGTCCATCTAGGACAGTGTGTTACGTGCAGTGACAAACAGTACCTCCAAGGTGG |
| *Cd47* | Leukocyte surface antigen CD47 | protein that may be involved in regulating renal cell growth; overexpression is associated with Fe-NTA-induced renal damage and tumor progression and metastasis | ENSRNOG00000001964 | qRnoCID0007645 | 96 | 94 | TCCTGTGGAGATTACAATGAGGCCAAGACCAGAAGCGTTCTTCACGGGCTTCTCTCCCGGGATGAAAAGGATGGCGCCAACAACCACGATGAGTGT |
| *Cd68* | Macrosialin precursor | plays a role in phagocytic activities of tissue macrophages, both in intracellular lysosomal metabolism and extracellular cell-cell and cell-pathogen interactions | ENSRNOG00000037563 | qRnoCED0005201 | 101 | 98 | AATGAGGATGCTCCTTGGTGGCCTACAGAGTGGACTGGAGCAAATGCTCAGAGGGGCTGGTAGGTTGATTGTCGTCTCCGGGTAACGCAGAAGGCAATGAG |
| *Cd8a* | T-cell surface glycoprotein CD8 alpha chain | increased expression on mast cells is induced by nitric oxide; may play a role in inflammatory response | ENSRNOG00000007178 | qRnoCED0001354 | 106 | 94 | GGATGCTCTTGGCTCTTCCGGAACTCCAGCTCCGAACTCCTCCAGCCCACCTTCATCATCTATGTATCTTCATCCCGGAGCAAGCTGAACGATATACTGGATCCGA |
| *Clec7a* | C-type lectin domain family 7 member A | necessary for the TLR2-mediated inflammatory response and activation of NF-kappa-B | ENSRNOG00000033879 | qRnoCED0008240 | 70 | 101 | GGTGGCATTAATGGACAATCTTAGAAGGCAGCAAGGCAGGTCACACTAGGAAACAGAGGCACATTCCAGT |
| *Csf1r* | Macrophage colony-stimulating factor 1 receptor precursor | may mediate normal and neoplastic growth of muscular cells | ENSRNOG00000018414 | qRnoCID0009152 | 93 | 95 | ATGTCTCTGCTGGTACTACTGCTGCTGCTGCTCTTGTACAAGTACAAGCAGAAGCCGAAATATCAGGTGCGCTGGAAGATCATTGAGAGCTAC |
| *Csf2* | Granulocyte-macrophage colony-stimulating factor | plays a role in alveolar epithelial fluid transport | ENSRNOG00000026805 | qRnoCED0004359 | 92 | 103 | GCAGTTCGTCTGGTAGTGGCTGGCTATCATGGTCAAGGCGCCATTGAGTTTGGTGAGGTTGCCCCGTAGACCCTGCTTGTATAGCTTCAGGC |
| *Csf3* | Granulocyte colony-stimulating factor precursor | putative hematopoetic growth factor for neutrophils | ENSRNOG00000008525 | qRnoCED0001885 | 117 | 98 | GCACTATGGTCAGGACAAGAGGCCATTCCCCTGCTCACCGTCAGCTCTCTGCCACCATCCCTGCCTTTGCCCCGAAGCTTTCTGCTTAAGTCCTTGGAGCAAGTGAGGAAGATTCAG |
| *Cxcl2* | C-X-C motif chemokine 2 | chemokine involved in the pulmonary inflammatory response | ENSRNOG00000002792 | qRnoCED0003624 | 102 | 99 | GCTGACTGAACACATTGAACATTATTACAATAAACTTCAACATATTAAAATGACCTCTTAAGATACTACAGTGAGCTGGCCAATGCATATCTTTAAATATCA |
| *Cxcl5* | C-X-C motif chemokine 5 precursor | member of the CXC chemokine family, is a neutrophil chemoattractant and is rapidly induced in response to muscle injury | ENSRNOG00000002843 | qRnoCED0002198 | 72 | 99 | TGAATGACTTTCTTTATCAACGGAGCTTCTGGGTCAAGACAAACATTATCCTTCTGGTTCTTCAACTTAGCT |
| *Cxcr2* | C-X-C chemokine receptor type 2 | chemokine receptor that binds Il8, GRO/MGSA and neutrophil activating peptide-2 | ENSRNOG00000014269 | qRnoCED0002513 | 116 | 101 | ACCGTAGTCTGCTATGAGAATATAGGTAATAATACATCCAAGTGGAGGGTGGTACTGCGCATCCTGCCTCAGACCTATGGCTTCCTCCTGCCGCTGCTCATCATGCTGTTCTGCTA |
| *Elane* | Neutrophil elastase precursor | Modifies the functions of natural killer cells, monocytes and granulocytes. | ENSRNOG00000033685 | qRnoCED0005566 | 64 | 100 | GGGCCTTAGTTGGTCCTGCCCTCTCTCTCTCGGTCTTTGGGATTGGTAAGTGGCCGGTCATCAT |
| *Il10* | Interleukin-10 | factor involved in the inhibition of cytokine synthesis | ENSRNOG00000004647 | qRnoCID0005930 | 80 | 98 | CCTTCAGTCAAGTGAAGACTTTCTTTCAAAAGAAGGACCAGCTGGACAACATACTGCTGACAGATTCCTTACTGCAGGAC |
| *Il1a* | Interleukin-1 alpha precursor | proinflammatory cytokine | ENSRNOG00000004575 | qRnoCID0002952 | 111 | 102 | ATGACCTGGAGGCCATAGCCCATGATTTAGAAGAGACCATCCAACCCAGATCAGCACCTCACAGCTTCCAGAATAATTTGAGATACAAATTGATAAGGATCGTCAAGCAGG |
| *Il4* | Interleukin-4 | Th2-type cytokine; may be involved in inflammatory response in eosinophils | ENSRNOG00000007624 | qRnoCID0002254 | 109 | 98 | GAGCGTGGACTCATTCACGGTGCAGCTTCTCAGTGAGTTCAGACCGCTGACACCTCTACAGAGTTTCCTCAGTTCACCGAGAACCCCAGACTTGTTCTTCAAGCACGGA |
| *Il6* | Interleukin-6 | a cytokine involved in development and possibly in neurodegenerative processes | ENSRNOG00000010278 | qRnoCID0053166 | 120 | 94 | CAGACCAGTATATACCACTTCACAAGTCGGAGGCTTAATTACATATGTTCTCAGGGAGATCTTGGAAATGAGAAAAGAGTTGTGCAATGGCAATTCTGATTGTATGAACAGCGATGATGC |
| *Itgam* | Integrin alpha-M precursor | integrin alpha subunit that interacts with integrin beta 2 subunit to form Mac-1 | ENSRNOG00000019728 | qRnoCID0002800 | 120 | 94 | TGCGTGTCAAGAAGAAGTAGTCAAAAACAAGGATGCTGGGGAGGTCAGAGTCTGCCTCCATGTCCGCAAGAACACCAAGGACAGGCTGCGAGAAGGAGACATCCAGAGCACTGTCACTTA |
| *Itgax* | Protein Itgax | mediates cell-cell interaction during inflammatory responses | ENSRNOG00000036703 | qRnoCID0002524 | 110 | 98 | CAGGTTGGACTATGGTGATGTCATCCCCATGGCGGAGGCTGCAGGCATCATCCGTTATGCAATTGGGGTAGGACAGGCCTTTTACCAAGCACAGTCCAGGCAAGAATTAA |
| *Itgb1* | Integrin beta-1 | beta subunit of integrin receptor which binds collagen | ENSRNOG00000010966 | qRnoCID0002035 | 115 | 100 | TGTGTATACAATGAGCCATTACTATGATTATCCTTCAATTGCTCACCTTGTTCAGAAACTAAGTGAAAATAATATTCAGACGATTTTTGCAGTCACTGAAGAGTTCCAGCCTGTT |
| *Mpo* | Myeloperoxidase precursor | myeloperoxidase family member that may contribute to hyperoxia-mediated lung injury via nitration of proteins, resulting in reactive nitrogen species | ENSRNOG00000008310 | qRnoCED0008317 | 69 | 98 | TGTTCTTAGACACGGTAGTGATGCCAGTGTTGTCACAGATGATGCGGGGCAAGGAGATGGTAGCCAAGG |
| *Mrc1* | Macrophage mannose receptor 1 precursor | mediates the endocytosis of glycoproteins by macrophages. | ENSRNOG00000018251 | qRnoCID0003739 | 73 | 92 | GAGATTCACGAGCAGATGTACCTCACAGGATTGACCAGTTCCTTGACCTCTGGACTCTGGATTGGACTCAACA |
| *Nos2* | Not Available | cytokine-inducible enzmyme involved in nitric oxide (NO) production | ENSRNOG00000049980 | qRnoCID0004849 | 109 | 101 | GATGCTTGTGACTCTTAGGGTCATCCTGTGTTGTTGGGCTGGGAATAGCACCTGGGGTTTTCTCCACGTTGTTGTTAATGTCCTTTTCCTCTTTCAGGTCACCTTGGTA |
| *Pax7* | Paired box protein Pax-7 | transcription factor that is involved in the regulation of muscle stem cells proliferation; plays a role in myogenesis and muscle regeneration. | ENSRNOG00000018739 | qRnoCED0004970 | 61 | 101 | GTCTCCACAGGAAGAAGTCCCAGCACCGCGGAGTGTTCCCCGAGCTTCATACGGCGCTGTG |
| *Pdgfra* | Platelet-derived growth factor receptor alpha precursor | acts as a receptor tyrosine kinase for PDGF; may play a role in glial cell generation | ENSRNOG00000002244 | qRnoCID0003041 | 96 | 102 | GTATAATGGCCGCTGTCTTCTTCCTTAGCCCGGATCAGCTTTAATTTGCTTTGATACCTCGTCTCCTGGCTCCTCTGCACATCGGTGGTGATCTCA |
| *Pecam1* | Platelet endothelial cell adhesion molecule | cell adhesion molecule, also intracellular signaling molecule; may mediate leukocyte adhesion and migration, angiogenesis, and thrombosis | ENSRNOG00000043391 | qRnoCED0009632 | 103 | 98 | ACACTGGTATTCCATGTCTCTGGTGGGCTTGTCTGTGAATGTCACTGGGTCATTGGAGTTCTTCTGGACGGTCTGGAAGTTGCTCTTTGCTCTTAGGAGGCGG |
| *Ppia* | Peptidyl-prolyl cis-trans isomerase A | may play a role in protein folding or intracellular protein transport | ENSRNOG00000006423 | qRnoCID0056995 | 106 | 102 | GCAGACATGGTCAACCCCACCGTGTTCTTCGACATCACGGCTGATGGCGAGCCCTTGGGTCGCGTCTGCTTCGAGCTGTTTGCAGACAAAGTTCCAAAGACAGCAG |
| *Ptprc* | Receptor-type tyrosine-protein phosphatase C | member of a family of heavily glycosylated leukocyte cell surface glycoproteins; displays extensive O-glycosylation | ENSRNOG00000000655 | qRnoCID0004326 | 120 | 101 | CTCTCTTTGCTCATCTCTAGTTCATGCTTAAGTGGCACTCTGTTAAAGTCATATGGAACAACGTTAGAACTCCTGTTTTTCTTTTTATTTTCTTCTTGATTTCCAATGTGCTGTGTCCTC |
| *RPLP2* | Not Available | plays an important role in the elongation step of protein synthesis | ENSRNOG00000037607 | qRnoCED0015635 | 60 | 89 | CGCTACGTTGCCTCTTATCTGCTGGCCGCCCTCGGGGGCAACTCCAATCCCAGCGCCAAA |
| *Rplp0* | 60S acidic ribosomal protein P0 | member of the ribosomal protein family; has similarity to other P ribosomal proteins over the carboxyl terminal sequence | ENSRNOG00000001148 | qRnoCED0005242 | 97 | 100 | TAAGCAGGCTGACTTGGTGTGAGGGGCTTAGTCGAAGAGACCGAATCCCATGTCCTCATCGGATTCCTCCGACTCTTCCTTTGCTTCGACCTTGGCT |
| *Stat1* | Signal transducer and activator of transcription 1 isoform alpha | component of the IFN-gamma receptor signaling pathway and other signaling pathways; plays a role in development of cytokine resistance | ENSRNOG00000014079 | qRnoCID0001659 | 99 | 99 | AGTCATATTCATCTTGTAAGTCTTCTAGGGTCTTGATTTCATGCTCTATGCACATGACTTGGTCCTTCACATTTCTGACTTTACTGTCCAGCTCCTTCT |
| *Stat3* | Signal transducer and activator of transcription 3 | transcription factor that plays a role in induction of gene expression during acute phase response | ENSRNOG00000019742 | qRnoCID0006149 | 100 | 98 | CTATACTGCTGGTCGATCTCGCCCAAGAGGTTATGAAACACCAGAGTGGCGTGTGACTCTTTGCTGGCTGCATATGCCCAATCTTGGCTCTCAATCCAAG |
| *Tek* | TEK tyrosine kinase, endothelial | mediates development of embryonic vasculature; plays a role in tumors and skin wounds | ENSRNOG00000008587 | qRnoCID0003531 | 76 | 98 | GACACCATCCGAACATCATTAATCTCTTGGGAGCATGTGAACACAGAGGCTACTTATACCTGGCTATTGAGTATGC |
| *Tgfb1* | Transforming growth factor beta-1 Latency-associated peptide | binds the TGFb receptor; plays a role in regulation of cell growth and proliferation; induces synthesis of extracellular matrix proteins and may play a role in fibrosis | ENSRNOG00000020652 | qRnoCID0009191 | 109 | 99 | GACGTCACTGGAGTTGTCCGGCAGTGGCTGAACCAAGGAGACGGAATACAGGGCTTTCGCTTCAGTGCTCACTGCTCTTGTGACAGCAAAGATAATGTACTCCACGTGG |
| *Tlr4* | Toll-like receptor 4 precursor | receptor that functions as the major upstream sensor for hemorrhagic shock and lipopolysaccharide | ENSRNOG00000010522 | qRnoCED0002945 | 117 | 102 | GGTCTAGAAGAGCTGGAATACCTGGACTTTCAGCACTCCACTTTAAAAAAGGTCACAGAATTCTCAGTGTTCTTATCTCTTGAAAAACTTCTTTACCTTGACATCTCTTACACTAAT |
| *Tlr9* | Toll-like receptor 9 precursor | Description Not Available | ENSRNOG00000048161 | qRnoCED0009119 | 67 | 99 | CTCAGCCATAACATCCTCAAGACTGTGGATCGCTCCTGGTTTGGGCCCATTGTGATGAACCTGACGG |
| *Vcam1* | Vascular cell adhesion protein 1 | binds integrin very late antigen (VLA4); plays a role in cell adhesion | ENSRNOG00000014333 | qRnoCID0005077 | 79 | 104 | GGAAGACTGTAAGCTGTATGTCCTTCGAAGAGCCTTGGATAATCAGTTCAACTGATTTTTTGCTAATTCCAGCCTCATT |
